# Exploring Options for Syndromic Surveillance in Aquaculture: Outbreak Detection of Salmon Pancreas Disease Using Production Data from Norwegian Farms

**DOI:** 10.1155/2024/9861677

**Published:** 2024-04-30

**Authors:** Victor H. S. Oliveira, Fernanda C. Dórea, Katharine R. Dean, Britt Bang Jensen

**Affiliations:** ^1^Norwegian Veterinary Institute, Ås N-1431, Oslo, Norway; ^2^Department of Disease Control and Epidemiology, National Veterinary Institute (SVA), Uppsala, SE-751 89, Sweden

## Abstract

Syndromic surveillance (SyS) is an important tool for early warning and monitoring of health in human and animal populations, but its use in aquaculture has been limited. Our study objective was to design a SyS system for Atlantic salmon aquaculture and to evaluate its performance in detecting pancreas disease (PD) outbreaks caused by salmonid alphaviruses on farms. We defined SyS outbreak alarms as cases where monthly farm mortality exceeded predefined cutoffs or deviated significantly from expected values based on predictive generalized linear models. These models were trained for each salmon production area in Norway, using data from 2014 to 2017. The outcome variable was fish mortality per farm-month, and input variables were production and environmental predictors, as well as an offset for the number of fish at risk. We also added autoregressive components to explain temporal dependency within fish cohorts. Subsequently, data from 2018 to 2021 was used to parameterize and validate the SyS system's performance against the current national PD surveillance program, which relies on routine farm-screening tests using molecular techniques and reports of clinical findings. The study covered 19,119 farm-months, involving 1,618 fish cohorts. The performance of our SyS system varied across production areas, with sensitivity ranging from 80.5% to 87.4% and a false alarm rate of 45.3%–53.2%. The absence of alarms was usually observed in farms that were truly negative for PD, i.e., a negative predictive value range of 81.2%–94.0%. The median time for alarms being raised was either in the same month as the current PD surveillance program or 1 month prior or after it. Our results indicate that the SyS system is a valuable tool for monitoring mortality on salmon farms, but alarms are unspecific if evaluated against an individual disease (PD). Increasing the frequency and granularity of mortality reporting might improve the SyS system's performance.

## 1. Introduction

Veterinary syndromic surveillance (SyS) utilizes both historical and current data, aiming to provide timely indications of health threats to animals. SyS involves leveraging preexisting data, often collected for other purposes, such as clinical, laboratory, and other diagnostic investigations. With these types of data, statistical methods are applied to detect aberrations in time series, considering geographical aspects and providing early warnings for potential disease outbreaks [1–3]. Being complementary to other traditional disease surveillance methods, SyS is also important for developing control measures and determining the need for interventions to mitigate risks of disease spread, especially when the impact on animal populations is significant. Although early SyS initiatives have concentrated on monitoring data of non-specific clinical syndromes, hence the terminology, other manifestations of poor health have also been widely used, including reduced production performance [4, 5] and mortality [6–9]. Veterinary SyS has primarily focused on terrestrial animals, including horses [10, 11], poultry [12, 13], cattle [4, 6, 8], small ruminants [14, 15], and swine [5, 7, 9]. In aquatic animals, the early detection of epidemics in shrimp farms has been evaluated based on production performance data, when there was reduced shrimp yield and survival [16]. Another study utilized mortality surveillance records to detect diseases in farmed salmon [17].

Norway is the world leader in Atlantic salmon aquaculture [18]. There has been an upward trend in the yearly number of harvested salmon, which reached more than 1.5 million tons in 2021, accompanied by plans for expansion [19, 20]. High salmon mortality is a challenge for the sustainability of such an expansion. Between 2017 and 2021, the annual mortality during the sea phase of salmon production (typically lasting 14–18 months) ranged from 15% to 18% in Norway [20]. The main single cause of mortality in captive salmon is injury due to increased handling during treatments. Together with infectious diseases, this accounts for almost half of the deaths [21]. The treatments generally target salmon lice (*Lepeophtheirus salmonis*) infestations, and they involve temporarily placing the salmon in heated water (thermal delousing) or mechanically removing the lice through brushing and flushing procedures. Among the infectious diseases affecting salmon in grow-out farms is pancreas disease (PD), caused by different genotypes of the salmon PD virus and commonly referred to as salmonid alphavirus (SAV). Salmon mortality after a PD diagnosis varies between baseline levels, below 1%, and up to 60% [22, 23]. Reduced growth of fish is another major problem related to PD [22, 24]. In 2013, a single Norwegian farm with 1 million fish and a PD outbreak had associated economic losses estimated at NOK 55.4 million (∼ € 7 million) [25]. Thus, not surprisingly, PD is a listed disease by the World Organization for Animal Health and notifiable at the national level, being endemic among farms located across several of the salmon production areas of Norway [20, 26]. In addition, an active surveillance program for PD was established in 2017, where all farms are screened for SAV by PCR monthly [27].

A characteristic of intensive salmon farming is an accumulation of data in production management systems, including mortality data. There are descriptions of mortality patterns in salmon farms, as well as regional, seasonal, and between farm variations [28, 29]. The national coverage of this data goes back to 2002, making it a good candidate for use as a health indicator in SyS. Additional data collected on production conditions and indicators such as environmental temperature and salinity, fish weight, applied treatments, and other fish management activities has been used to study factors associated with salmon mortality [29, 30]. Therefore, these data represent potentially useful information for the detection of deviations from expected mortality patterns and the detection of aberrations, which can be used as outbreak signals. The objective of this study was to design a SyS system in Atlantic salmon aquaculture using mortality data and to evaluate its performance in detecting PD outbreaks on farms using data from the national surveillance program.

## 2. Materials and Methods

### 2.1. Data

The proposed SyS system is based on data routinely collected for other purposes.  Table 1 presents an overview of these data, together with its reporting frequency and accessibility. The Norwegian Directorate of Fisheries maintains updated information about the geographical location of farms and types of farm licenses in a publicly available register. Each aquaculture facility has a unique identification number, which we utilized to combine with the other datasets. Farmers are obliged to submit production data to the Norwegian Directorate of Fisheries regarding counts of live, dead, and other losses of fish, stocking month, and mean fish weight at farms [31]. These data are not publicly available, but the Norwegian Veterinary Institute (NVI), as the National reference laboratory for animal diseases, can use them for disease control purposes. The Norwegian Food Safety Authority (NFSA) acquires data from farmers related to sea surface temperature and salmon lice treatments, which can be bath treatments using warm water, freshwater, H_2_O_2_, medicinal compounds (such as azamethiphos and pyrethroids), and removal of sea lice by flushing or brushing. Records of PD outbreaks on farms are derived from a combination of clinical investigations conducted by field veterinarians and fish inspectors, alongside molecular and pathological findings associated with PD, in samples processed at both private and national reference laboratories [27]. The PD data are directly reported and stored in databases at NVI. The described data from NFSA and confirmed PD outbreaks from NVI are openly accessible.

**Figure 1:**
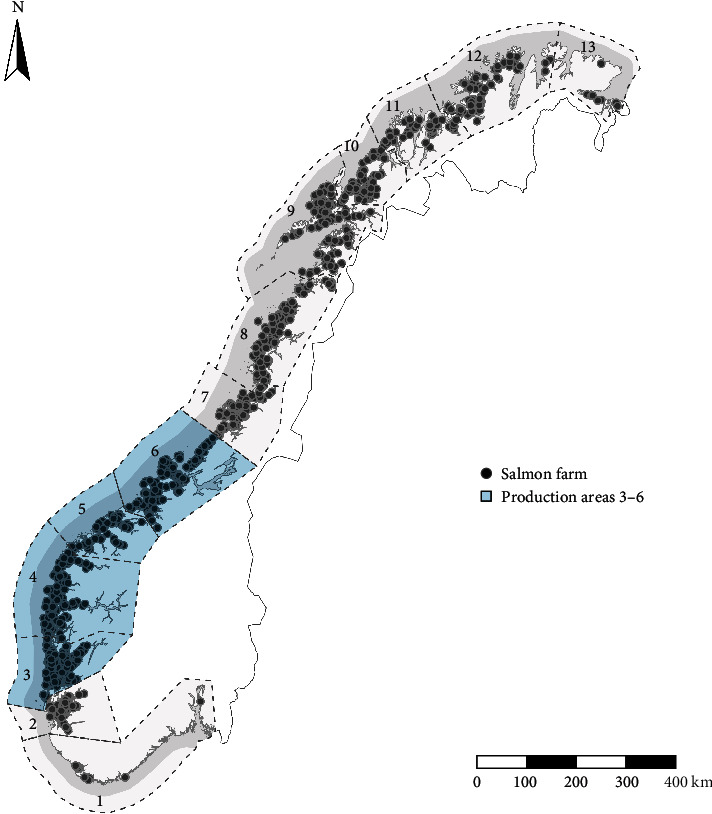
Location of the 13 production areas and farms licensed to produce Atlantic salmon for food consumption between 2014 and 2021 in Norway. This study considered the production areas 3−6, highlighted in blue, where PD is endemic.

We used data from all marine farms that are licensed to produce Atlantic salmon for food consumption in the production areas 3−6 (out of 13) of the Norwegian coast (Figure 1). Farms in these areas are within the boundaries where PD is endemic [27]. We excluded production months with salmon weighing less than 400 g as they are rarely affected by PD. Although fish in this weight class have a high overall mortality, it is most likely due to seawater adaptation of post-smolts [21, 22, 24]. We also excluded data from months with salmon weighing more than 6 kg or farms with salmon stocked for more than 24 continuous months. These data do not represent a typical production cycle in Norwegian grow-out salmon farms. We processed and analyzed the data using R statistical software version 4.2.2 [32].

### 2.2. Syndromic Salmon Mortality Surveillance System

Salmon mortality was the syndromic indicator of the surveillance system. We calculated the monthly mortality rate (MR) in every farm using Equation (1).

(1) $$\begin{array}{c} \text{MR}=\frac{deaths}{\left(start-\frac{deaths}{2}-\frac{wth}{2}+\frac{add}{2}\right)\times time}, \end{array}$$

where *deaths* is the number of dead salmon, *start* is the initial number of live fish, *wth* is the number of withdrawn salmon from the farm due to losses other than deaths, *add* is the number of added fish to the farm, and *time* is 1 month. The denominator of this equation represents the number of fish units at risk during 1 month [29, 33].

We designed the SyS system using an expanding window method. A training set with a predefined period expands over a more recent dataset in which we implemented alarm statistics. The alarm statistics were generated monthly per farm without dropping older data. Implementation of the method for the training set started in January 2014. The alarm statistics period, which also includes progressively added data for the training set, started in January 2018 and ended in December 2021.

**Figure 2:**
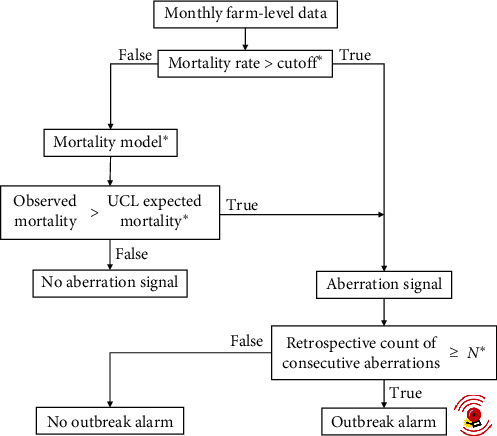
Workflow for the syndromic salmon mortality surveillance system. Parameters indicated by the asterisks can be adjusted to improve system performance. UCL = upper control limit and *N* = number of consecutive months with aberrations.

In the SyS system, there were two pathways for the detection of an aberration signal, as represented in Figure 2. First, farms with mortality in a month above a fixed cutoff were treated as non-baseline mortality cases and classified as aberrations (not yet an outbreak alarm). Second, for farms with mortality below the cutoff, we compared the mortality observed in the month with mortality estimated by a model fitted with data gathered up to the previous month, as described below. Finally, we set up alarms for a farm corresponding to potential disease outbreaks when a certain number of consecutive aberrations had occurred (“*N*” in Figure 2).

### 2.3. Model for Baseline Mortality

During the model-building process, we excluded data from the non-baseline aberrations and from cases with inconsistencies in the recorded number of fish, primarily arising from discrepancies in the reported numbers of deaths and other losses from the previous month. The analysis proceeded with individual models fitted for each production area due to the known variation in mortality patterns between these areas [28, 29]. We fitted negative-binomial generalized linear models using the “glm.nb()” function of the MASS package [34], with individual fish deaths for each farm-month as the outcome, along with an offset for the number of fish at risk. We used temperature, fish weight, salmon lice treatments, stocking month, and fish weight upon stocking as explanatory variables, all of which have been shown to be associated with salmon mortality [29]. The models also included auto-regressive components corresponding to the observed number of fish deaths in the previous month to account for temporal dependency within each cohort. Based on the entire training period, the model makes a prediction for the mortality in each farm-cohort for the next month. An upper control limit (UCL) for that prediction is calculated using the “qnbinom()” function in R to obtain the desired quantile of a negative binomial distribution with the predicted value as mean and a shape parameter (theta) estimated from the fitted model [35]. Finally, the observed mortality for the corresponding month is compared to the UCL and observed values above the UCL are flagged as aberrations. As the model is applied prospectively, a month forward at a time, values flagged as aberrations are not incorporated into the baseline. Aberrations are instead replaced with the UCL for that farm-month as previously described [36].

### 2.4. Parametrization

We performed parametrization of the SyS system using multiple settings for the parameters indicated by asterisks in Figure 2. For the MR cutoffs related to non-baseline aberrations, we tested values between 0.3% and 5% in increments of 0.1%. These values represent a range of overall mortality observed in Norwegian aquaculture since the beginning of the study period, excluding outliers [28, 29]. For parameterization of the mortality models, we used a constant model as in Model I of Table 2, without independent variables, and different models with variables associated with mortality [29], using Models II–VIII in Table 2. The tested UCL corresponded to mortality values in the sequence between the 10th and 90th quantiles in increments of five. The number of consecutive months with aberrations ranged from one to six, representing the typical duration of PD outbreaks [22, 24]. When testing different values for a parameter, we kept the values of the remaining parameters fixed as follows: mortality rate cutoff of 2%, mortality model VI (Table 2), 65th quantile for the UCL, and three consecutive aberrations for generating an outbreak alarm (“*N*” in Figure 2).

### 2.5. Performance Assessment

We evaluated the ability of the proposed SyS system to detect PD outbreaks monthly by comparing it to the current PD surveillance program outputs. These evaluations were conducted from January 2018 (just a few months after the initiation of Norway's current national screening program for PD) to December 2021. The screening program is based on a regulation that mandates all marine farms with a standing stock of salmonids, not known to be infected with PD, to submit samples from 20 fish each month to an approved laboratory for SAV detection by polymerase chain reaction [27].

**Figure 3:**
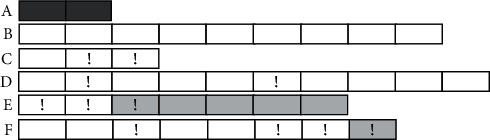
Schematic representation of production months and fish cohorts considered positive or negative for pancreas disease (PD) using the proposed syndromic surveillance system for outbreak alarms. In this example, an outbreak alarm requires three consecutive months with aberration signals. Each rectangle represents one production month, and the sequential rectangles represent farms with fish cohorts A–F. Cohort A had fish stocked for less than 3 months and was ineligible for outbreak generation, represented by dark gray rectangles. Cohort B, shown with all empty rectangles, did not exhibit any aberration signals or trigger outbreak alarms. Cohorts C and D had aberration signals, represented by exclamation marks, but they did not meet the criteria for outbreak alarms. Conversely, Cohorts E and F had PD outbreak alarms, represented by light gray rectangles after three consecutive months with aberration signals. Cohort E and F were positive for PD during five and one production months, respectively, as indicated by the number of light gray rectangles representing the alarms. In summary, Cohorts B, C, and D had no alarms for PD outbreaks in any of the production months and were therefore classified as negative PD cohorts. On the other hand, Cohorts E and F had monthly alarms for PD outbreaks and were classified as positive PD cohorts.

In addition to the monthly evaluations, we evaluated the PD status of a farm at the cohort level. A cohort was a group of fish on a farm followed from the first stocking month until the evaluation month with stocking information and no gaps corresponding to periods of inactivity on the farm. In both the SyS system and the PD surveillance program, a farm is considered infected and thus maintains a PD outbreak status from the month PD is first detected until the slaughtering month.  Figure 3 presents examples of production months and cohorts that are positive and negative for PD outbreaks according to the proposed SyS system. For illustration purposes, the system settings in these examples required the detection of three consecutive aberrations to trigger an outbreak alarm, either through mortality cutoffs or predictive models. However, several settings were tested during the system's parametrization.

With the results of the detection of PD outbreaks by the proposed SyS system and the current surveillance program, we calculated performance measures based on the following categorization: true positive (TP), which was PD positive by both the SyS system and the surveillance program; false positive (FP), which were PD positive by the SyS system and PD negative by the surveillance program; true negative (TN), which were negative by both the SyS system and the surveillance program; and false negative (FN), which were PD negative by the SyS system and PD positive by the surveillance program. The performance measures for evaluating the system included sensitivity (Se), *Se*=TP/(TP+FN); false alarm rate (FAR), FAR=FP/(FP+TN); positive predictive value (PPV), PPV=TP/(TP+FP); and negative predictive value (NPV), NPV=TN/(TN+FN). We calculated monthly-level metrics by comparing the monthly results from the SyS system with those of the PD surveillance program and summarizing the results. The same was done at the cohort level. We also evaluated cohort-level timeliness as the difference between the month of PD detection by the SyS system and the current surveillance program. We used tables with the calculated performance measures for comparisons of the implementation of the SyS system under multiple settings and across production areas.

## 3. Results

The data analyzed in this study initially had records from 433 farms in Norway in production areas 3−6, including 1,660 fish cohorts and 23,213 production months. After applying the exclusion criteria (explained in the subheading “Data” of this article), we retained data from 19,119 production months and 1,618 fish cohorts, which were stocked in 430 salmon farms.  Table 3 presents these data stratified by production area for the full study period (2014−2021). Additionally, we describe the data for the alarm statistics period (2018–2021), which excludes the training period at the beginning of the dataset. This specific period corresponds to what we use to evaluate the SyS system's performance. During the alarm statistics period, the fish cohorts experiencing PD outbreaks numbered 86 in area 3, 95 in area 4, 23 in area 5, and 109 in area 6. These figures do not represent the overall prevalence of PD in Norway due to our specific exclusion criteria.

In Table 4, we present the parameters that demonstrated the best performance in detecting PD outbreaks, providing a reasonable balance between high sensitivity and low FAR when comparing different performance measures with varying parameters. In Table 5, we show the performance measures obtained after running the SyS system with such parameters. See *Supplementary 1* and *2* for the SyS system's results with other parameter values.

The monthly-based SyS system with the best performance had aberrations detected, with MR cutoffs varying between 0.3% and 0.5% across the production areas. In cases where aberrations were detected through predictive models, these models had, in addition to the autoregressive component, only temperature as the independent variable (model IV) for area 4, or temperature, fish weight, and salmon lice treatments (model VI) for production areas 3, 5, and 6. The UCLs in these models were set using quantiles from 40% to 60%. The number of consecutive aberrations to generate alarms was three for all the production areas. With the selected settings, we found a high sensitivity of the SyS system, over 80% in all the production areas, with the highest sensitivity in production area 3 at 87.4%. The FAR ranged from 45.3% to 53.2% across the production areas. The PPV was highest in production area 4, indicating a 50.6% probability of PD among the production months with alarms. Conversely, production area 5 had the lowest PPV at 32.5%. The high FAR and low PPV demonstrate that the high sensitivity was achieved at the cost of specificity. Notably, this translated into high NPVs in all the production areas, for example, 94% in production area 3, demonstrates the system's ability to correctly identify farms without PD in most cases.

In contrast to the monthly results, at the cohort level, it was necessary to set higher MR cutoffs (2.1%−2.5%) to achieve satisfactory performance of the SyS system. In production areas 3, 5, and 6, we selected model V with the autoregressive component, temperature, and weight as explanatory variables, while in production area 4, we observed better results with model VII, which included sea lice treatments and stocking month as additional variables. The UCLs for aberration detection were also higher for each production area compared to the monthly evaluations, ranging from 0.65 to 0.75. We used two consecutive aberrations for triggering alarms in production areas 5 and 6 and three for production areas 3 and 4. The balance of sensitivity against the FAR was somewhat similar in production areas 3, 4, and 6, considering the sensitivity results equal to or larger than 80%, and the FAR range was 46.3%−51.5%. As opposed to this, the results for production area 5 differed considerably, as achieving a sensitivity close to 80% while maintaining a low FAR was not possible (*Supplementary 1* and *2*). PPVs were better in production areas 4 and 6, where approximately two out of three cohorts with PD outbreaks could be detected by the SyS system. In contrast, only half of the outbreaks in production area 3 and one-third in production area 5 could be detected. NPVs were lower in the cohort-level evaluations but still high for production area 3 (87%). However, for the other production areas, there was a more relevant drop of the NPVs, as exemplified by production area 5, which had the worst NPV, with a 64.3% probability of a cohort with no alarms being free from PD. Overall, the median timeliness of detecting PD outbreaks by the SyS system was comparable to the current PD surveillance program. The first outbreak alarms for a cohort occurred in the same month, in the preceding or following month.

Implementation of the SyS system, both monthly and at the cohort level, can be adjusted if there is interest in prioritizing one of the performance measures over others (*Supplementary 1* and *2*). For example, adjusting the settings with larger MR cutoffs, higher UCLs from the models, not using any model, or more aberrations necessary to raise outbreak alarms decreased the FAR but with substantial negative effects on the sensitivity of the system. Such adjustments increased the PPV and reduced the NPV, albeit to a lesser extent.

## 4. Discussion

We provided a health monitoring SyS based on fish production data, which had to be uniquely designed to fit the production structure. The design of our SyS system was based on previously documented methodologies used for disease early warning systems in animal and human health. The literature concerning SyS systems in aquatic animals is scarce when compared to that available for terrestrial animals. It is important to consider the variations in production types, baseline mortality patterns, and disease outbreak characteristics between distinct species and the specific characteristics of aquaculture production. These factors have an influence on the design and performance of SyS systems.

An endemic disease (PD) was used to explore the use of SyS in salmon aquaculture, specifically chosen due to its notable occurrence of outbreaks within the production areas under investigation. Therefore, our study presents a way to prospectively validate a SyS system. This type of validation would become more difficult if reliant on data from exotic or sporadic diseases (e.g., infectious salmon anemia surveillance, which also involves routine data collection in Norway [37]). The difficulty arises from the lack of outbreaks in a certain population or the limited number of disease outbreaks within the study period. As a common strategy for testing SyS systems, others employed simulations to create scenarios that can approximate the characteristics of real disease outbreaks [5, 38, 39].

Training data were generated using a combination of methods to remove potential aberration signals in historical data. Typically, when building SyS systems, data preprocessing is recommended to remove high-magnitude aberrations from the syndromic time series [2, 35]. These aberrational data would not represent the onset of an adverse health event but rather the course of the event, which is primarily not targeted by an early warning system. In previous research, the historical baseline data has been defined after the removal of extreme data points after visual inspections of time series [40]. In some studies, the preprocessing consisted of removing data associated with known disease outbreaks or positive laboratory results for pathogens [41–44]. Other studies determined cutoffs for the removal of aberrational data based on quantiles of a fitted model using the whole dataset, and that can account, for instance, for seasonality or other response variables [36, 43, 45]. By combining the initial step of removing extreme mortality from the data, followed by using model-defined cutoffs, we achieved better system performance compared to a single approach (see *Supplementary 1*). The combined approach resulted in achieving a dataset with a closer representation of mortality at baseline levels than those otherwise acquired, which is important to optimize subsequent predictions by SyS models [43].

The prospective detection of aberrations in the aquaculture production systems offered unique challenges due to the structure of the animal population aggregated in farms and in production cohorts that are at different production stages at any given time. Both the structural and temporal correlations had to be specifically accounted for when devising an algorithm that could compare observed mortality values with expected ones. Previous research adopted autoregressive integrated moving average models [8, 46] or the Holt–Winters forecasting method to deal with temporal correlation. In cases where temporal correlation could be ignored or removed, control charts such as Shewhart charts, exponentially weighted moving average charts, and cumulative sum charts were common options of aberration detection for SyS systems [5, 12, 47–51]. However, these methods were less suitable for our system since we could not assume the constancy of the analyzed time series. The quantity and length of salmon production cycles on farms vary depending on market demand, the size of fish put to sea, as well as environmental and regulatory factors [52, 53]. The fact that our system was designed to trigger alarms for farms, instead of at the regional or national level, was also an obstacle to applying the other methods. A fish cohort usually lasts less than 24 months (about 2 years), and capturing meaningful patterns in addition to variations in these time series that could be useful for estimating model parameters and establishing control limits for the charts would be challenging. Although previous studies in pigs [5] and poultry [12] described SyS systems using control charts with production data, they analyzed data on a weekly or daily basis. The length of our time series observations, per fish cohort, may decrease if farmers choose to shorten the production cycle period at sea by the production of larger smolts in freshwater before the transfer. This management procedure has recently been implemented by some farmers to minimize fish exposure to diseases [52]. To address the issue of a few monthly observations to analyze, we leveraged the data of predictors associated with mortality from many cohorts to estimate model parameters. Temperature, for example, was included in our final models for each production area and served as a proxy for accounting for seasonality. We still accounted for the autoregressive behavior in the short-length time series, considering the presence of lagged deaths in the final models.

The integration of consecutive months with aberrations before raising outbreak alarms resembles strategies previously applied to prevent excessive detection of unusual events [54, 55], which could mostly represent isolated cases and not actual outbreaks. The suitability of such an approach, when evaluating the SyS system performance against PD, lies in the fact that PD does not generally affect fish in the first few months after sea transfer [22]. This suitability is further supported by a study indicating that increased mortality due to PD precedes clinical outbreak detection and persists at elevated levels until fish slaughtering [30]. If the goal is to identify potential health problems in the early stage after sea transfer of salmon (i.e., the first 2–3 months), our approach is inadequate due to the frequency of the time series. This applies to diseases that typically occur in recently transferred fish, for example, tenacibaculosis, infectious pancreatic necrosis and mortality due to incomplete smoltification [20, 21, 56]. It is important to note that these diseases affecting recently transferred fish are likely to have been carried from the freshwater phase, as a certain exposure time is necessary for horizontal transmission from surrounding farms in the sea. The availability of monthly data on fish mortality during the freshwater phase presents an opportunity for further investigations that integrate fish from hatcheries into the SyS system. However, the feasible use of this data is hindered by the lack of proper identification of fish that belongs to specific cohorts, which arises from common practices in hatcheries involving grading and movement of fish between tanks [57]. For diseases that can occur at any point during seawater production, alarm delays are a consequence in younger fish. Complex gill disease, winter ulcer, heart and skeletal muscle inflammation, and cardiomyopathy syndrome are a good illustration of such diseases, which were pointed out by fish health personnel and inspectors as the main causes of mortality in grow-out salmon farms [20].

With respect to aspects of our system's performance, the variations in the PPV and NPV characterize how these measures are not solely determined by the system being tested [33] but are also influenced by the PD prevalences that differ across the production areas [20]. Additionally, it may be argued that the outcome of SyS is influenced by several determinant factors, including the pathogen's ability to spread between and within animal groups, as well as the impact of the associated disease on the syndromic indicator [4, 58]. Specifically, for PD, the severity of clinical manifestations that result in mortality varies according to the associated subtype of SAV [59]. While our SyS system successfully identified most of the PD outbreaks, it was accompanied by numerous false alarms. This raises the question of whether potential users of the system, such as farmers or competent authorities, would disregard the alarms. However, it is noteworthy that the performance test was only evaluated against an individual disease (PD), and thus, other diseases were likely associated with the increased mortality detected by the system, resulting in false positives for PD. This discussion can be extended to the timeliness aspect of SyS. Both early and delayed alarms associated with PD could be due to other health threats involved in single or mixed infections, but that did not take part in the SyS performance evaluations. Data regarding the main non-notifiable diseases in Norwegian farmed salmon exists with the producers [20], and, despite not being readily accessible, it could be a source of information for additional performance tests depending on data access agreements. Due to the nature of SyS systems, which use unspecific health-related data, they cannot definitively pinpoint specific diseases on farms. Follow-up investigations are necessary to determine the actual cause of disease outbreaks [60, 61].

The analyses flow created is highly customizable. We evaluated several different parameterization options, not only within the statistical model, but also including different decision points regarding high mortality and a number of aberrations to generate an alarm. As highlighted above, the performance was only evaluated against PD detection, but this high customization potential would allow the system to be tested for other diseases and, most importantly, as a non-specific indicator of health, flagging farms that may need more attention. The performance evaluation showed that this was a highly sensitive but low-specificity health monitoring tool, which can, therefore, be used as a first screening in combination with other surveillance methods.

Supporting the usefulness of our system in decision-making processes and on-farm management is the need for farmers and other stakeholders to benchmark mortality on a single or neighboring farm rather than generalizing to larger geographical areas. Salmon farming stakeholders expressed the importance of the availability of such information in a timely manner in a qualitative study (Zhou, personal communication, September 2023). Informed decisions could be made that are important to maintain high biosecurity status and to mitigate risks of disease introductions on farms. For example, implementing controls over the contact network between farms (e.g., wellboat movements used for transportation, handling, and sorting of live fish), devising vaccination strategies, and determining the optimized duration of fallowing periods [24, 62–64].

There is significant potential for progress in SyS within salmon aquaculture. The acquisition of the data with higher time granularity, building upon existing datasets, would enable us to run the system more frequently, at least on a weekly basis, thereby promoting timeliness in detecting aberrations. We designed the proposed SyS system using only mortality series, as opposed to several studies that adopted multivariate approaches, i.e., using time series from multiple health-related indicators, to trigger the alarms [5, 10, 40]. Studies demonstrated performance improvements with the multivariate SyS systems [10], as well as when applying Bayesian frameworks to these types of datasets [65, 66]. Additional data sources that may be worth further exploration for SyS include sensor data [67, 68]. For instance, this involves measurements of water parameters such as dissolved oxygen levels and turbidity. Imaging data can also be utilized for monitoring fish behavior. Particularly for a system built using mortality information, there is an ongoing initiative within the salmon industry to collect data with assigned causes of death [21, 69]. The primary data stream on mortality is divided into different mortality classifications, each focusing on specific causes of death, including assigned deaths due to infectious diseases, physiological causes, traumatic incidents, and environmental factors. Integrating these separate data streams into the SyS system could be beneficial. However, to date, this data has less coverage and concentrates on certain production areas. Additionally, a meticulous validation process is crucial to ensure that the data from multiple sources, enriched with mortality information on each farm, is consistent enough to be effectively utilized for detecting aberrations [70].

## 5. Conclusions

The proposed SyS system demonstrated high sensitivity and reasonable timeliness in detecting outbreaks of an endemic disease (PD) in salmon farms, but it was unspecific when evaluated against this single disease. For most months, when farms were free from PD, our system did not generate alarms. These findings have the potential to inform and guide revisions to the current PD surveillance program, which imposes significant costs associated with the large-scale sampling, as well as the labor and personnel required for the investigations. Integration of the SyS system into the strategies adopted in the current program in a sequential manner might be an option to transition towards a more risk-based program that maintains comparable performance with reduced overall program costs. The system required adaptation of the utilized models per production area to achieve optimized performance. Improvements in the system's performance may be possible by incorporating data with higher resolution in the time series and more granularity to the mortality information, as well as adding datasets of other syndromic indicators. Overall, the SyS system successfully generated alarms that were useful for monitoring mortality associated with health threats to farmed salmon populations. Thus, it holds great potential as a supportive tool for farmers and stakeholders, enabling them to make informed decisions concerning on-farm management and disease prevention. This, in turn, may lead to enhanced animal welfare and increased productivity within salmon farming operations. Additionally, competent authorities could use the SyS system as a valuable resource for response planning in animal health surveillance initiatives.

## Figures and Tables

**Table 1:** Overview of data used for developing and evaluating the performance of a syndromic surveillance system in aquaculture.

Data type	Description	Scale	Report frequency	Accessibility
Farm ID	Unique identification of farms	Categorical	Not applicable	Open^†^
Production area	Production areas 1–13 established by regulation	Categorical	Not applicable	Open^†^
Farm license	Type of farm license, including species and purpose of production	Categorical	On-demand	Open^†^
Fish counts	Number of stocked fish	Discrete	Monthly	Closed
Fish deaths	Number of dead fish	Discrete	Monthly	Closed
Fish losses	Number of fish losses other than deaths, such as discarded and escaped fish	Discrete	Monthly	Closed
Fish weight	Mean weight of fish in grams	Continuous	Monthly	Closed
Temperature	Average sea surface temperature in Celsius	Continuous	Weekly	Open^‡^
Salmon lice treatments	Number and type of treatments applied to control salmon lice	Discrete	Weekly	Open^‡^
PD outbreak	Suspected or not suspected pancreas disease outbreak	Dichotomous	Daily	Open^‡^

^†^ https://www.fiskeridir.no/Akvakultur/Registre-og-skjema/akvakulturregisteret. ^‡^https://www.barentswatch.no/fiskehelse.

**Table 2:** Fitted models used for parametrization of the proposed syndromic surveillance system.

Model	Equation
I	$\begin{array}{c} \log\;\left(\text{deaths}\right)=\alpha +\varepsilon \end{array}$
II	$\begin{array}{c} \log\;\left(\text{deaths}\right)=\alpha +\log\;\left(\text{fish at risk during 1 month}\right)+\varepsilon \end{array}$
III	$\begin{array}{c} \log\;\left(\text{deaths}\right)=\alpha +\beta_{1}\text{ lagged deaths }+\log\;\left(\text{fish at risk during 1 month}\right)+\varepsilon \end{array}$
IV	$\begin{array}{c} \log\;\left(\text{deaths}\right)=\alpha +\beta_{1}\text{ lagged deaths }+\beta_{2}\text{ temperature}+\log\;\left(\text{fish at risk during 1 month}\right)+\varepsilon \end{array}$
V	$\begin{array}{c} \log\;\left(\text{deaths}\right)=\alpha +\beta_{1}\text{ lagged deaths }+\beta_{2}\text{ temperature}+\beta_{3}\text{ weight}+\log\;\left(\text{fish at risk during 1 month}\right)+\varepsilon \end{array}$
VI	$\begin{array}{c} \log\;\left(\text{deaths}\right)=\alpha +\beta_{1}\text{ lagged deaths }+\beta_{2}\text{ temperature}+\beta_{3}\text{ weight}+\beta_{4}\text{ salmon lice treatments }+\log\;\left(\text{fish at risk during 1 month}\right)+\varepsilon \end{array}$
VII	$\begin{array}{c} \log\;\left(\text{deaths}\right)=\alpha +\beta_{1}\text{ lagged deaths }+\beta_{2}\text{ temperature}+\beta_{3}\text{ weight}+\beta_{4}\text{ salmon lice treatments }+\beta_{5}\text{ stocking month}+\\ \log\;\left(\text{fish at risk during 1 month}\right)+\varepsilon \end{array}$
VIII	$\begin{array}{c} \log\;\left(\text{deaths}\right)=\alpha +\beta_{1}\text{ lagged deaths }+\beta_{2}\text{ temperature}+\beta_{3}\text{ weight}+\beta_{4}\text{ salmon lice treatments }+\beta_{5}\text{ stocking month}+\\ \beta_{6}\text{ weight upon stocking}+\log\;\left(\text{fish at risk during 1 month}\right)+\varepsilon \end{array}$

Aberration detection of the system included comparisons of observed mortality in salmon farms with estimated mortality by models.

**Table 3:** Number of farms, fish cohorts, and production months used to design and test the performance of a syndromic salmon mortality surveillance system.

Area	Full study period, years 2014−2021 (alarm statistics period, years 2018−2021)		
	Farms	Cohorts	Months
3	137 (130)	576 (328)	6,597 (3,278)

4	122 (115)	427 (251)	4,842 (2,444)

5	40 (39)	138 (85)	1,760 (934)

6	131 (122)	477 (281)	5,920 (3,050)

All	430 (406)	1,618 (945)	19,119 (9,706)

The numbers are stratified by production areas in Norwegian coastal waters for different study periods.

**Table 4:** Parameters of the proposed syndromic surveillance (SyS) system using mortality data from salmon farms in Norway.

Production area	Parameter			
	MR cutoff (%)	Model	UCL	*N* aberrations
Monthly SyS				
3	0.5	Model VI	0.55	3
4	0.3	Model IV	0.6	3
5	0.5	Model VI	0.55	3
6	0.4	Model VI	0.4	3
Cohort SyS				
3	2.3	Model V	0.65	3
4	2.3	Model VII	0.65	3
5	2.1	Model V	0.7	2
6	2.5	Model V	0.75	2

We selected parameters for the implementation of the SyS system monthly and at the cohort level as well across the different production areas in Norwegian coastal waters. The models are detailed in Table 2. *Abbreviations*. MR = mortality rate, UCL = upper control limit, and *N* aberrations = number of consecutive aberrations detected for an alarm.

**Table 5:** Performance of the proposed syndromic surveillance system using mortality data in detecting salmon pancreas disease outbreaks monthly and at cohort level.

Area	Monthly performance^†^				Cohort performance^†^			
	3	4	5	6	3	4	5	6
TP	500	582	147	629	74	76	18	89
FP	993	569	306	917	69	37	33	51
TN	1,123	688	281	808	80	38	9	48
FN	72	107	34	152	12	19	5	20
Se	87.4%	84.5%	81.2%	80.5%	86.0%	80.0%	78.3%	81.7%
FAR	46.9%	45.3%	52.1%	53.2%	46.3%	49.3%	78.6%	51.5%
PPV	33.5%	50.6%	32.5%	40.7%	51.7%	67.3%	35.3%	63.6%
NPV	94.0%	86.5%	89.2%	84.2%	87.0%	66.7%	64.3%	70.6%
Time^‡^	—	—	—	—	1 (−3 to 4)	1 (−2 to 3)	−1 (−3 to 1)	0 (−4 to 4)

The values in this table refer to the system implemented using the parameters presented in Table 4. TP = true positive, FP = false positive, TN = true negative, FN = false negative, Se = sensitivity, FAR = false alarm rate, PPV = positive predictive value, NPV = negative predictive value, and Time = timeliness. ^†^Sum of TP, FP, TN, and FN is not always equal to the numbers given in Table 3 due to a minimum number of months with aberration detection required to trigger outbreak alarms. ^‡^Time is given as the median (interquartile range) of the difference between the month of PD detection by the SyS system and the current surveillance program.

## Data Availability

Some of the data used to support the findings of this study are publicly available online; please see Table 1 for the sources. Data used for mapping can be retrieved from http://www.kartverket.no. Other production data cannot be made available because of privacy agreements; details of these cases are specified in Table 1. The R software code used in this study is also available (https://doi.org/10.5281/zenodo.8401684).

## Supplementary Materials

Supplementary 1 Results of the monthly performance evaluation of the proposed syndromic surveillance system using various parameter values. TP = true positive, FP = false positive, TN = true negative, FN = false negative, Se = sensitivity, FAR = false alarm rate, PPV = positive predictive value, NPV = negative predictive value.

Supplementary 2 Results of the cohorts' performance evaluation of the proposed syndromic surveillance system using various parameter values. TP = true positive, FP = false positive, TN = true negative, FN = false negative, Se = sensitivity, FAR = false alarm rate, PPV = positive predictive value, NPV = negative predictive value, time = timeliness, Q1 = 1st quantile, Q3 = 3rd quantile.
