# Supplementary material for: Exploring Options for Syndromic Surveillance in Aquaculture: Outbreak Detection of Salmon Pancreas Disease Using Production Data from Norwegian Farms
Source: Transbound Emerg Dis. 2024 Apr 30;2024:9861677. doi: 10.1155/2024/9861677 (PMC12017065; doi:10.1155/2024/9861677)
Supplement: Supplementary 1 — Results of the monthly performance evaluation of the proposed syndromic surveillance system using various parameter values. TP = true positive, FP = false positive, TN = true negative, FN = false negative, Se = sensitivity, FAR = false alarm rate, PPV = positive predictive value, NPV = negative predictive value. [file 9861677.f1.pdf]

Parameter	parameter_setting	Zone	TP	FP	TN	FN	FAR	TPR(Se)	TNR(Sp)	PPV	NPV
baseline.correction	FALSE	3	504	998	1118	68	0.4716	0.8811	0.5284	0.3356	0.9427
baseline.correction	TRUE	3	510	1048	1068	62	0.4953	0.8916	0.5047	0.3273	0.9451
Model	1	3	511	1152	964	61	0.5444	0.8934	0.4556	0.3073	0.9405
Model	2	3	516	1052	1064	56	0.4972	0.9021	0.5028	0.3291	0.9500
Model	3	3	516	1049	1067	56	0.4957	0.9021	0.5043	0.3297	0.9501
Model	4	3	515	1048	1068	57	0.4953	0.9003	0.5047	0.3295	0.9493
Model	5	3	510	1048	1068	62	0.4953	0.8916	0.5047	0.3273	0.9451
Model	6	3	510	1048	1068	62	0.4953	0.8916	0.5047	0.3273	0.9451
Model	7	3	507	1047	1069	65	0.4948	0.8864	0.5052	0.3263	0.9427
Model	8	3	507	1047	1069	65	0.4948	0.8864	0.5052	0.3263	0.9427
Model	no_model	3	444	588	1528	128	0.2779	0.7762	0.7221	0.4302	0.9227
MR_cutoff	0.003	3	530	1215	901	42	0.5742	0.9266	0.4258	0.3037	0.9555
MR_cutoff	0.004	3	517	1113	1003	55	0.5260	0.9038	0.4740	0.3172	0.9480
MR_cutoff	0.005	3	510	1048	1068	62	0.4953	0.8916	0.5047	0.3273	0.9451
MR_cutoff	0.006	3	497	980	1136	75	0.4631	0.8689	0.5369	0.3365	0.9381
MR_cutoff	0.007	3	488	913	1203	84	0.4315	0.8531	0.5685	0.3483	0.9347
MR_cutoff	0.008	3	476	867	1249	96	0.4097	0.8322	0.5903	0.3544	0.9286
MR_cutoff	0.009	3	455	821	1295	117	0.3880	0.7955	0.6120	0.3566	0.9171
MR_cutoff	0.01	3	442	766	1350	130	0.3620	0.7727	0.6380	0.3659	0.9122
MR_cutoff	0.011	3	431	742	1374	141	0.3507	0.7535	0.6493	0.3674	0.9069
MR_cutoff	0.012	3	414	710	1406	158	0.3355	0.7238	0.6645	0.3683	0.8990
MR_cutoff	0.013	3	405	691	1425	167	0.3266	0.7080	0.6734	0.3695	0.8951
MR_cutoff	0.014	3	395	668	1448	177	0.3157	0.6906	0.6843	0.3716	0.8911
MR_cutoff	0.015	3	380	649	1467	192	0.3067	0.6643	0.6933	0.3693	0.8843
MR_cutoff	0.016	3	361	627	1489	211	0.2963	0.6311	0.7037	0.3654	0.8759
MR_cutoff	0.017	3	354	608	1508	218	0.2873	0.6189	0.7127	0.3680	0.8737
MR_cutoff	0.018	3	346	582	1534	226	0.2750	0.6049	0.7250	0.3728	0.8716
MR_cutoff	0.019	3	343	569	1547	229	0.2689	0.5997	0.7311	0.3761	0.8711
MR_cutoff	0.02	3	339	561	1555	233	0.2651	0.5927	0.7349	0.3767	0.8697
MR_cutoff	0.021	3	329	546	1570	243	0.2580	0.5752	0.7420	0.3760	0.8660
MR_cutoff	0.022	3	325	541	1575	247	0.2557	0.5682	0.7443	0.3753	0.8644
MR_cutoff	0.023	3	322	531	1585	250	0.2509	0.5629	0.7491	0.3775	0.8638
MR_cutoff	0.024	3	312	509	1607	260	0.2405	0.5455	0.7595	0.3800	0.8607
MR_cutoff	0.025	3	299	501	1615	273	0.2368	0.5227	0.7632	0.3738	0.8554

MR_cutoff	0.026	3	301	492	1624	271	0.2325	0.5262	0.7675	0.3796	0.8570
MR_cutoff	0.027	3	298	486	1630	274	0.2297	0.5210	0.7703	0.3801	0.8561
MR_cutoff	0.028	3	294	476	1640	278	0.2250	0.5140	0.7750	0.3818	0.8551
MR_cutoff	0.029	3	281	458	1658	291	0.2164	0.4913	0.7836	0.3802	0.8507
MR_cutoff	0.03	3	279	444	1672	293	0.2098	0.4878	0.7902	0.3859	0.8509
MR_cutoff	0.031	3	282	437	1679	290	0.2065	0.4930	0.7935	0.3922	0.8527
MR_cutoff	0.032	3	272	434	1682	300	0.2051	0.4755	0.7949	0.3853	0.8486
MR_cutoff	0.033	3	268	432	1684	304	0.2042	0.4685	0.7958	0.3829	0.8471
MR_cutoff	0.034	3	263	419	1697	309	0.1980	0.4598	0.8020	0.3856	0.8460
MR_cutoff	0.035	3	259	421	1695	313	0.1990	0.4528	0.8010	0.3809	0.8441
MR_cutoff	0.036	3	259	406	1710	313	0.1919	0.4528	0.8081	0.3895	0.8453
MR_cutoff	0.037	3	259	397	1719	313	0.1876	0.4528	0.8124	0.3948	0.8460
MR_cutoff	0.038	3	253	396	1720	319	0.1871	0.4423	0.8129	0.3898	0.8436
MR_cutoff	0.039	3	250	389	1727	322	0.1838	0.4371	0.8162	0.3912	0.8429
MR_cutoff	0.04	3	250	386	1730	322	0.1824	0.4371	0.8176	0.3931	0.8431
MR_cutoff	0.041	3	248	385	1731	324	0.1819	0.4336	0.8181	0.3918	0.8423
MR_cutoff	0.042	3	247	384	1732	325	0.1815	0.4318	0.8185	0.3914	0.8420
MR_cutoff	0.043	3	246	381	1735	326	0.1801	0.4301	0.8199	0.3923	0.8418
MR_cutoff	0.044	3	242	380	1736	330	0.1796	0.4231	0.8204	0.3891	0.8403
MR_cutoff	0.045	3	241	376	1740	331	0.1777	0.4213	0.8223	0.3906	0.8402
MR_cutoff	0.046	3	238	380	1736	334	0.1796	0.4161	0.8204	0.3851	0.8386
MR_cutoff	0.047	3	244	379	1737	328	0.1791	0.4266	0.8209	0.3917	0.8412
MR_cutoff	0.048	3	238	379	1737	334	0.1791	0.4161	0.8209	0.3857	0.8387
MR_cutoff	0.049	3	238	379	1737	334	0.1791	0.4161	0.8209	0.3857	0.8387
MR_cutoff	0.05	3	240	375	1741	332	0.1772	0.4196	0.8228	0.3902	0.8398
N_aberrations	1	3	553	1697	451	19	0.7900	0.9668	0.2100	0.2458	0.9596
N_aberrations	2	3	534	1328	812	38	0.6206	0.9336	0.3794	0.2868	0.9553
N_aberrations	3	3	510	1048	1068	62	0.4953	0.8916	0.5047	0.3273	0.9451
N_aberrations	4	3	476	835	1245	93	0.4014	0.8366	0.5986	0.3631	0.9305
N_aberrations	5	3	434	657	1387	135	0.3214	0.7627	0.6786	0.3978	0.9113
N_aberrations	6	3	385	517	1487	184	0.2580	0.6766	0.7420	0.4268	0.8899
UCL	0.05	3	560	1639	477	12	0.7746	0.9790	0.2254	0.2547	0.9755
UCL	0.1	3	558	1621	495	14	0.7661	0.9755	0.2339	0.2561	0.9725
UCL	0.15	3	557	1573	543	15	0.7434	0.9738	0.2566	0.2615	0.9731
UCL	0.2	3	554	1487	629	18	0.7027	0.9685	0.2973	0.2714	0.9722

UCL	0.25	3	547	1385	731	25	0.6545	0.9563	0.3455	0.2831	0.9669
UCL	0.3	3	538	1284	832	34	0.6068	0.9406	0.3932	0.2953	0.9607
UCL	0.35	3	529	1219	897	43	0.5761	0.9248	0.4239	0.3026	0.9543
UCL	0.4	3	527	1152	964	45	0.5444	0.9213	0.4556	0.3139	0.9554
UCL	0.45	3	513	1083	1033	59	0.5118	0.8969	0.4882	0.3214	0.9460
UCL	0.5	3	510	1048	1068	62	0.4953	0.8916	0.5047	0.3273	0.9451
UCL	0.55	3	500	993	1123	72	0.4693	0.8741	0.5307	0.3349	0.9397
UCL	0.6	3	488	927	1189	84	0.4381	0.8531	0.5619	0.3449	0.9340
UCL	0.65	3	476	858	1258	96	0.4055	0.8322	0.5945	0.3568	0.9291
UCL	0.7	3	458	780	1336	114	0.3686	0.8007	0.6314	0.3700	0.9214
UCL	0.75	3	441	717	1399	131	0.3388	0.7710	0.6612	0.3808	0.9144
UCL	0.8	3	426	645	1471	146	0.3048	0.7448	0.6952	0.3978	0.9097
UCL	0.85	3	411	577	1539	161	0.2727	0.7185	0.7273	0.4160	0.9053
UCL	0.9	3	394	535	1581	178	0.2528	0.6888	0.7472	0.4241	0.8988
UCL	0.95	3	387	521	1595	185	0.2462	0.6766	0.7538	0.4262	0.8961
baseline.correction	FALSE	4	583	576	681	106	0.4582	0.8462	0.5418	0.5030	0.8653
baseline.correction	TRUE	4	588	601	656	101	0.4781	0.8534	0.5219	0.4945	0.8666
Model	1	4	568	611	646	121	0.4861	0.8244	0.5139	0.4818	0.8422
Model	2	4	595	606	651	94	0.4821	0.8636	0.5179	0.4954	0.8738
Model	3	4	591	607	650	98	0.4829	0.8578	0.5171	0.4933	0.8690
Model	4	4	590	596	661	99	0.4741	0.8563	0.5259	0.4975	0.8697
Model	5	4	588	601	656	101	0.4781	0.8534	0.5219	0.4945	0.8666
Model	6	4	588	601	656	101	0.4781	0.8534	0.5219	0.4945	0.8666
Model	7	4	590	595	662	99	0.4733	0.8563	0.5267	0.4979	0.8699
Model	8	4	590	593	664	99	0.4718	0.8563	0.5282	0.4987	0.8702
Model	no_model	4	509	353	904	180	0.2808	0.7388	0.7192	0.5905	0.8339
MR_cutoff	0.003	4	614	701	556	75	0.5577	0.8911	0.4423	0.4669	0.8811
MR_cutoff	0.004	4	599	642	615	90	0.5107	0.8694	0.4893	0.4827	0.8723
MR_cutoff	0.005	4	588	601	656	101	0.4781	0.8534	0.5219	0.4945	0.8666
MR_cutoff	0.006	4	575	567	690	114	0.4511	0.8345	0.5489	0.5035	0.8582
MR_cutoff	0.007	4	564	539	718	125	0.4288	0.8186	0.5712	0.5113	0.8517
MR_cutoff	0.008	4	545	526	731	144	0.4185	0.7910	0.5815	0.5089	0.8354
MR_cutoff	0.009	4	540	507	750	149	0.4033	0.7837	0.5967	0.5158	0.8343
MR_cutoff	0.01	4	523	476	781	166	0.3787	0.7591	0.6213	0.5235	0.8247
MR_cutoff	0.011	4	515	462	795	174	0.3675	0.7475	0.6325	0.5271	0.8204

MR_cutoff	0.012	4	505	445	812	184	0.3540	0.7329	0.6460	0.5316	0.8153
MR_cutoff	0.013	4	493	421	836	196	0.3349	0.7155	0.6651	0.5394	0.8101
MR_cutoff	0.014	4	488	416	841	201	0.3309	0.7083	0.6691	0.5398	0.8071
MR_cutoff	0.015	4	477	404	853	212	0.3214	0.6923	0.6786	0.5414	0.8009
MR_cutoff	0.016	4	469	392	865	220	0.3119	0.6807	0.6881	0.5447	0.7972
MR_cutoff	0.017	4	463	384	873	226	0.3055	0.6720	0.6945	0.5466	0.7944
MR_cutoff	0.018	4	444	370	887	245	0.2944	0.6444	0.7056	0.5455	0.7836
MR_cutoff	0.019	4	432	365	892	257	0.2904	0.6270	0.7096	0.5420	0.7763
MR_cutoff	0.02	4	417	357	900	272	0.2840	0.6052	0.7160	0.5388	0.7679
MR_cutoff	0.021	4	410	352	905	279	0.2800	0.5951	0.7200	0.5381	0.7644
MR_cutoff	0.022	4	405	348	909	284	0.2768	0.5878	0.7232	0.5378	0.7619
MR_cutoff	0.023	4	406	345	912	283	0.2745	0.5893	0.7255	0.5406	0.7632
MR_cutoff	0.024	4	402	339	918	287	0.2697	0.5835	0.7303	0.5425	0.7618
MR_cutoff	0.025	4	398	340	917	291	0.2705	0.5776	0.7295	0.5393	0.7591
MR_cutoff	0.026	4	379	325	932	310	0.2586	0.5501	0.7414	0.5384	0.7504
MR_cutoff	0.027	4	369	322	935	320	0.2562	0.5356	0.7438	0.5340	0.7450
MR_cutoff	0.028	4	362	313	944	327	0.2490	0.5254	0.7510	0.5363	0.7427
MR_cutoff	0.029	4	359	309	948	330	0.2458	0.5210	0.7542	0.5374	0.7418
MR_cutoff	0.03	4	353	304	953	336	0.2418	0.5123	0.7582	0.5373	0.7393
MR_cutoff	0.031	4	351	303	954	338	0.2411	0.5094	0.7589	0.5367	0.7384
MR_cutoff	0.032	4	346	298	959	343	0.2371	0.5022	0.7629	0.5373	0.7366
MR_cutoff	0.033	4	345	296	961	344	0.2355	0.5007	0.7645	0.5382	0.7364
MR_cutoff	0.034	4	343	297	960	346	0.2363	0.4978	0.7637	0.5359	0.7351
MR_cutoff	0.035	4	341	294	963	348	0.2339	0.4949	0.7661	0.5370	0.7346
MR_cutoff	0.036	4	341	291	966	348	0.2315	0.4949	0.7685	0.5396	0.7352
MR_cutoff	0.037	4	337	287	970	352	0.2283	0.4891	0.7717	0.5401	0.7337
MR_cutoff	0.038	4	337	282	975	352	0.2243	0.4891	0.7757	0.5444	0.7347
MR_cutoff	0.039	4	335	280	977	354	0.2228	0.4862	0.7772	0.5447	0.7340
MR_cutoff	0.04	4	334	278	979	355	0.2212	0.4848	0.7788	0.5458	0.7339
MR_cutoff	0.041	4	330	278	979	359	0.2212	0.4790	0.7788	0.5428	0.7317
MR_cutoff	0.042	4	323	269	988	366	0.2140	0.4688	0.7860	0.5456	0.7297
MR_cutoff	0.043	4	322	269	988	367	0.2140	0.4673	0.7860	0.5448	0.7292
MR_cutoff	0.044	4	321	267	990	368	0.2124	0.4659	0.7876	0.5459	0.7290
MR_cutoff	0.045	4	320	267	990	369	0.2124	0.4644	0.7876	0.5451	0.7285
MR_cutoff	0.046	4	316	264	993	373	0.2100	0.4586	0.7900	0.5448	0.7269

MR_cutoff	0.047	4	316	261	996	373	0.2076	0.4586	0.7924	0.5477	0.7275
MR_cutoff	0.048	4	312	260	997	377	0.2068	0.4528	0.7932	0.5455	0.7256
MR_cutoff	0.049	4	312	260	997	377	0.2068	0.4528	0.7932	0.5455	0.7256
MR_cutoff	0.05	4	312	259	998	377	0.2060	0.4528	0.7940	0.5464	0.7258
N_aberrations	1	4	662	1017	266	28	0.7927	0.9594	0.2073	0.3943	0.9048
N_aberrations	2	4	629	777	498	60	0.6094	0.9129	0.3906	0.4474	0.8925
N_aberrations	3	4	588	601	656	101	0.4781	0.8534	0.5219	0.4945	0.8666
N_aberrations	4	4	540	468	776	147	0.3762	0.7860	0.6238	0.5357	0.8407
N_aberrations	5	4	487	358	860	198	0.2939	0.7109	0.7061	0.5763	0.8129
N_aberrations	6	4	433	269	929	252	0.2245	0.6321	0.7755	0.6168	0.7866
UCL	0.05	4	663	934	323	26	0.7430	0.9623	0.2570	0.4152	0.9255
UCL	0.1	4	663	917	340	26	0.7295	0.9623	0.2705	0.4196	0.9290
UCL	0.15	4	663	879	378	26	0.6993	0.9623	0.3007	0.4300	0.9356
UCL	0.2	4	651	826	431	38	0.6571	0.9448	0.3429	0.4408	0.9190
UCL	0.25	4	637	788	469	52	0.6269	0.9245	0.3731	0.4470	0.9002
UCL	0.3	4	622	750	507	67	0.5967	0.9028	0.4033	0.4534	0.8833
UCL	0.35	4	613	703	554	76	0.5593	0.8897	0.4407	0.4658	0.8794
UCL	0.4	4	600	665	592	89	0.5290	0.8708	0.4710	0.4743	0.8693
UCL	0.45	4	589	622	635	100	0.4948	0.8549	0.5052	0.4864	0.8639
UCL	0.5	4	588	601	656	101	0.4781	0.8534	0.5219	0.4945	0.8666
UCL	0.55	4	582	569	688	107	0.4527	0.8447	0.5473	0.5056	0.8654
UCL	0.6	4	562	543	714	127	0.4320	0.8157	0.5680	0.5086	0.8490
UCL	0.65	4	542	512	745	147	0.4073	0.7866	0.5927	0.5142	0.8352
UCL	0.7	4	529	480	777	160	0.3819	0.7678	0.6181	0.5243	0.8292
UCL	0.75	4	495	425	832	194	0.3381	0.7184	0.6619	0.5380	0.8109
UCL	0.8	4	474	387	870	215	0.3079	0.6880	0.6921	0.5505	0.8018
UCL	0.85	4	456	350	907	233	0.2784	0.6618	0.7216	0.5658	0.7956
UCL	0.9	4	443	319	938	246	0.2538	0.6430	0.7462	0.5814	0.7922
UCL	0.95	4	429	298	959	260	0.2371	0.6226	0.7629	0.5901	0.7867
baseline.correction	FALSE	5	133	275	312	48	0.4685	0.7348	0.5315	0.3260	0.8667
baseline.correction	TRUE	5	136	291	296	45	0.4957	0.7514	0.5043	0.3185	0.8680
Model	1	5	103	286	301	78	0.4872	0.5691	0.5128	0.2648	0.7942
Model	2	5	136	287	300	45	0.4889	0.7514	0.5111	0.3215	0.8696
Model	3	5	144	290	297	37	0.4940	0.7956	0.5060	0.3318	0.8892
Model	4	5	139	287	300	42	0.4889	0.7680	0.5111	0.3263	0.8772

Model	5	5	135	286	301	46	0.4872	0.7459	0.5128	0.3207	0.8674
Model	6	5	136	291	296	45	0.4957	0.7514	0.5043	0.3185	0.8680
Model	7	5	142	289	298	39	0.4923	0.7845	0.5077	0.3295	0.8843
Model	8	5	138	292	295	43	0.4974	0.7624	0.5026	0.3209	0.8728
Model	no_model	5	109	191	396	72	0.3254	0.6022	0.6746	0.3633	0.8462
MR_cutoff	0.003	5	154	328	259	27	0.5588	0.8508	0.4412	0.3195	0.9056
MR_cutoff	0.004	5	144	307	280	37	0.5230	0.7956	0.4770	0.3193	0.8833
MR_cutoff	0.005	5	136	291	296	45	0.4957	0.7514	0.5043	0.3185	0.8680
MR_cutoff	0.006	5	136	275	312	45	0.4685	0.7514	0.5315	0.3309	0.8739
MR_cutoff	0.007	5	127	270	317	54	0.4600	0.7017	0.5400	0.3199	0.8544
MR_cutoff	0.008	5	121	254	333	60	0.4327	0.6685	0.5673	0.3227	0.8473
MR_cutoff	0.009	5	117	244	343	64	0.4157	0.6464	0.5843	0.3241	0.8428
MR_cutoff	0.01	5	108	240	347	73	0.4089	0.5967	0.5911	0.3103	0.8262
MR_cutoff	0.011	5	108	234	353	73	0.3986	0.5967	0.6014	0.3158	0.8286
MR_cutoff	0.012	5	107	227	360	74	0.3867	0.5912	0.6133	0.3204	0.8295
MR_cutoff	0.013	5	106	220	367	75	0.3748	0.5856	0.6252	0.3252	0.8303
MR_cutoff	0.014	5	101	207	380	80	0.3526	0.5580	0.6474	0.3279	0.8261
MR_cutoff	0.015	5	100	203	384	81	0.3458	0.5525	0.6542	0.3300	0.8258
MR_cutoff	0.016	5	90	193	394	91	0.3288	0.4972	0.6712	0.3180	0.8124
MR_cutoff	0.017	5	89	189	398	92	0.3220	0.4917	0.6780	0.3201	0.8122
MR_cutoff	0.018	5	85	183	404	96	0.3118	0.4696	0.6882	0.3172	0.8080
MR_cutoff	0.019	5	83	168	419	98	0.2862	0.4586	0.7138	0.3307	0.8104
MR_cutoff	0.02	5	79	165	422	102	0.2811	0.4365	0.7189	0.3238	0.8053
MR_cutoff	0.021	5	73	156	431	108	0.2658	0.4033	0.7342	0.3188	0.7996
MR_cutoff	0.022	5	72	150	437	109	0.2555	0.3978	0.7445	0.3243	0.8004
MR_cutoff	0.023	5	75	151	436	106	0.2572	0.4144	0.7428	0.3319	0.8044
MR_cutoff	0.024	5	66	148	439	115	0.2521	0.3646	0.7479	0.3084	0.7924
MR_cutoff	0.025	5	66	145	442	115	0.2470	0.3646	0.7530	0.3128	0.7935
MR_cutoff	0.026	5	66	140	447	115	0.2385	0.3646	0.7615	0.3204	0.7954
MR_cutoff	0.027	5	65	139	448	116	0.2368	0.3591	0.7632	0.3186	0.7943
MR_cutoff	0.028	5	65	139	448	116	0.2368	0.3591	0.7632	0.3186	0.7943
MR_cutoff	0.029	5	63	137	450	118	0.2334	0.3481	0.7666	0.3150	0.7923
MR_cutoff	0.03	5	63	136	451	118	0.2317	0.3481	0.7683	0.3166	0.7926
MR_cutoff	0.031	5	63	136	451	118	0.2317	0.3481	0.7683	0.3166	0.7926
MR_cutoff	0.032	5	63	136	451	118	0.2317	0.3481	0.7683	0.3166	0.7926

MR_cutoff	0.033	5	60	132	455	121	0.2249	0.3315	0.7751	0.3125	0.7899
MR_cutoff	0.034	5	63	135	452	118	0.2300	0.3481	0.7700	0.3182	0.7930
MR_cutoff	0.035	5	61	134	453	120	0.2283	0.3370	0.7717	0.3128	0.7906
MR_cutoff	0.036	5	61	127	460	120	0.2164	0.3370	0.7836	0.3245	0.7931
MR_cutoff	0.037	5	63	130	457	118	0.2215	0.3481	0.7785	0.3264	0.7948
MR_cutoff	0.038	5	63	129	458	118	0.2198	0.3481	0.7802	0.3281	0.7951
MR_cutoff	0.039	5	59	130	457	122	0.2215	0.3260	0.7785	0.3122	0.7893
MR_cutoff	0.04	5	59	118	469	122	0.2010	0.3260	0.7990	0.3333	0.7936
MR_cutoff	0.041	5	59	118	469	122	0.2010	0.3260	0.7990	0.3333	0.7936
MR_cutoff	0.042	5	59	118	469	122	0.2010	0.3260	0.7990	0.3333	0.7936
MR_cutoff	0.043	5	59	118	469	122	0.2010	0.3260	0.7990	0.3333	0.7936
MR_cutoff	0.044	5	57	115	472	124	0.1959	0.3149	0.8041	0.3314	0.7919
MR_cutoff	0.045	5	57	113	474	124	0.1925	0.3149	0.8075	0.3353	0.7926
MR_cutoff	0.046	5	57	111	476	124	0.1891	0.3149	0.8109	0.3393	0.7933
MR_cutoff	0.047	5	57	110	477	124	0.1874	0.3149	0.8126	0.3413	0.7937
MR_cutoff	0.048	5	56	109	478	125	0.1857	0.3094	0.8143	0.3394	0.7927
MR_cutoff	0.049	5	56	110	477	125	0.1874	0.3094	0.8126	0.3373	0.7924
MR_cutoff	0.05	5	56	110	477	125	0.1874	0.3094	0.8126	0.3373	0.7924
N_aberrations	1	5	166	459	134	15	0.7740	0.9171	0.2260	0.2656	0.8993
N_aberrations	2	5	151	365	226	30	0.6176	0.8343	0.3824	0.2926	0.8828
N_aberrations	3	5	136	291	296	45	0.4957	0.7514	0.5043	0.3185	0.8680
N_aberrations	4	5	123	229	358	58	0.3901	0.6796	0.6099	0.3494	0.8606
N_aberrations	5	5	109	176	395	72	0.3082	0.6022	0.6918	0.3825	0.8458
N_aberrations	6	5	99	133	423	82	0.2392	0.5470	0.7608	0.4267	0.8376
UCL	0.05	5	174	463	124	7	0.7888	0.9613	0.2112	0.2732	0.9466
UCL	0.1	5	171	449	138	10	0.7649	0.9448	0.2351	0.2758	0.9324
UCL	0.15	5	168	415	172	13	0.7070	0.9282	0.2930	0.2882	0.9297
UCL	0.2	5	165	392	195	16	0.6678	0.9116	0.3322	0.2962	0.9242
UCL	0.25	5	161	365	222	20	0.6218	0.8895	0.3782	0.3061	0.9174
UCL	0.3	5	161	345	242	20	0.5877	0.8895	0.4123	0.3182	0.9237
UCL	0.35	5	154	332	255	27	0.5656	0.8508	0.4344	0.3169	0.9043
UCL	0.4	5	147	317	270	34	0.5400	0.8122	0.4600	0.3168	0.8882
UCL	0.45	5	143	306	281	38	0.5213	0.7901	0.4787	0.3185	0.8809
UCL	0.5	5	136	291	296	45	0.4957	0.7514	0.5043	0.3185	0.8680
UCL	0.55	5	133	275	312	48	0.4685	0.7348	0.5315	0.3260	0.8667

UCL	0.6	5	125	264	323	56	0.4497	0.6906	0.5503	0.3213	0.8522
UCL	0.65	5	115	248	339	66	0.4225	0.6354	0.5775	0.3168	0.8370
UCL	0.7	5	112	237	350	69	0.4037	0.6188	0.5963	0.3209	0.8353
UCL	0.75	5	103	224	363	78	0.3816	0.5691	0.6184	0.3150	0.8231
UCL	0.8	5	91	203	384	90	0.3458	0.5028	0.6542	0.3095	0.8101
UCL	0.85	5	82	190	397	99	0.3237	0.4530	0.6763	0.3015	0.8004
UCL	0.9	5	79	172	415	102	0.2930	0.4365	0.7070	0.3147	0.8027
UCL	0.95	5	69	159	428	112	0.2709	0.3812	0.7291	0.3026	0.7926
baseline.correction	FALSE	6	542	664	1061	239	0.3849	0.6940	0.6151	0.4494	0.8162
baseline.correction	TRUE	6	584	738	987	197	0.4278	0.7478	0.5722	0.4418	0.8336
Model	1	6	558	789	936	223	0.4574	0.7145	0.5426	0.4143	0.8076
Model	2	6	576	781	944	205	0.4528	0.7375	0.5472	0.4245	0.8216
Model	3	6	572	768	957	209	0.4452	0.7324	0.5548	0.4269	0.8208
Model	4	6	575	750	975	206	0.4348	0.7362	0.5652	0.4340	0.8256
Model	5	6	579	739	986	202	0.4284	0.7414	0.5716	0.4393	0.8300
Model	6	6	584	738	987	197	0.4278	0.7478	0.5722	0.4418	0.8336
Model	7	6	573	727	998	208	0.4214	0.7337	0.5786	0.4408	0.8275
Model	8	6	572	723	1002	209	0.4191	0.7324	0.5809	0.4417	0.8274
Model	no_model	6	429	355	1370	352	0.2058	0.5493	0.7942	0.5472	0.7956
MR_cutoff	0.003	6	620	896	829	161	0.5194	0.7939	0.4806	0.4090	0.8374
MR_cutoff	0.004	6	603	812	913	178	0.4707	0.7721	0.5293	0.4261	0.8368
MR_cutoff	0.005	6	584	738	987	197	0.4278	0.7478	0.5722	0.4418	0.8336
MR_cutoff	0.006	6	540	672	1053	241	0.3896	0.6914	0.6104	0.4455	0.8138
MR_cutoff	0.007	6	516	622	1103	265	0.3606	0.6607	0.6394	0.4534	0.8063
MR_cutoff	0.008	6	502	601	1124	279	0.3484	0.6428	0.6516	0.4551	0.8011
MR_cutoff	0.009	6	489	579	1146	292	0.3357	0.6261	0.6643	0.4579	0.7969
MR_cutoff	0.01	6	477	550	1175	304	0.3188	0.6108	0.6812	0.4645	0.7945
MR_cutoff	0.011	6	451	515	1210	330	0.2986	0.5775	0.7014	0.4669	0.7857
MR_cutoff	0.012	6	435	492	1233	346	0.2852	0.5570	0.7148	0.4693	0.7809
MR_cutoff	0.013	6	419	476	1249	362	0.2759	0.5365	0.7241	0.4682	0.7753
MR_cutoff	0.014	6	413	463	1262	368	0.2684	0.5288	0.7316	0.4715	0.7742
MR_cutoff	0.015	6	403	426	1299	378	0.2470	0.5160	0.7530	0.4861	0.7746
MR_cutoff	0.016	6	392	407	1318	389	0.2359	0.5019	0.7641	0.4906	0.7721
MR_cutoff	0.017	6	386	392	1333	395	0.2272	0.4942	0.7728	0.4961	0.7714
MR_cutoff	0.018	6	376	369	1356	405	0.2139	0.4814	0.7861	0.5047	0.7700

MR_cutoff	0.019	6	370	364	1361	411	0.2110	0.4738	0.7890	0.5041	0.7681
MR_cutoff	0.02	6	364	352	1373	417	0.2041	0.4661	0.7959	0.5084	0.7670
MR_cutoff	0.021	6	358	338	1387	423	0.1959	0.4584	0.8041	0.5144	0.7663
MR_cutoff	0.022	6	350	328	1397	431	0.1901	0.4481	0.8099	0.5162	0.7642
MR_cutoff	0.023	6	350	325	1400	431	0.1884	0.4481	0.8116	0.5185	0.7646
MR_cutoff	0.024	6	345	324	1401	436	0.1878	0.4417	0.8122	0.5157	0.7627
MR_cutoff	0.025	6	335	316	1409	446	0.1832	0.4289	0.8168	0.5146	0.7596
MR_cutoff	0.026	6	327	309	1416	454	0.1791	0.4187	0.8209	0.5142	0.7572
MR_cutoff	0.027	6	324	299	1426	457	0.1733	0.4149	0.8267	0.5201	0.7573
MR_cutoff	0.028	6	324	295	1430	457	0.1710	0.4149	0.8290	0.5234	0.7578
MR_cutoff	0.029	6	321	285	1440	460	0.1652	0.4110	0.8348	0.5297	0.7579
MR_cutoff	0.03	6	318	278	1447	463	0.1612	0.4072	0.8388	0.5336	0.7576
MR_cutoff	0.031	6	316	275	1450	465	0.1594	0.4046	0.8406	0.5347	0.7572
MR_cutoff	0.032	6	316	269	1456	465	0.1559	0.4046	0.8441	0.5402	0.7579
MR_cutoff	0.033	6	314	264	1461	467	0.1530	0.4020	0.8470	0.5433	0.7578
MR_cutoff	0.034	6	307	255	1470	474	0.1478	0.3931	0.8522	0.5463	0.7562
MR_cutoff	0.035	6	307	257	1468	474	0.1490	0.3931	0.8510	0.5443	0.7559
MR_cutoff	0.036	6	303	252	1473	478	0.1461	0.3880	0.8539	0.5459	0.7550
MR_cutoff	0.037	6	297	247	1478	484	0.1432	0.3803	0.8568	0.5460	0.7533
MR_cutoff	0.038	6	296	247	1478	485	0.1432	0.3790	0.8568	0.5451	0.7529
MR_cutoff	0.039	6	292	247	1478	489	0.1432	0.3739	0.8568	0.5417	0.7514
MR_cutoff	0.04	6	292	247	1478	489	0.1432	0.3739	0.8568	0.5417	0.7514
MR_cutoff	0.041	6	293	244	1481	488	0.1414	0.3752	0.8586	0.5456	0.7522
MR_cutoff	0.042	6	286	243	1482	495	0.1409	0.3662	0.8591	0.5406	0.7496
MR_cutoff	0.043	6	284	241	1484	497	0.1397	0.3636	0.8603	0.5410	0.7491
MR_cutoff	0.044	6	282	239	1486	499	0.1386	0.3611	0.8614	0.5413	0.7486
MR_cutoff	0.045	6	273	234	1491	508	0.1357	0.3496	0.8643	0.5385	0.7459
MR_cutoff	0.046	6	272	233	1492	509	0.1351	0.3483	0.8649	0.5386	0.7456
MR_cutoff	0.047	6	273	228	1497	508	0.1322	0.3496	0.8678	0.5449	0.7466
MR_cutoff	0.048	6	271	229	1496	510	0.1328	0.3470	0.8672	0.5420	0.7458
MR_cutoff	0.049	6	271	228	1497	510	0.1322	0.3470	0.8678	0.5431	0.7459
MR_cutoff	0.05	6	270	229	1496	511	0.1328	0.3457	0.8672	0.5411	0.7454
N_aberrations	1	6	708	1256	481	74	0.7231	0.9054	0.2769	0.3605	0.8667
N_aberrations	2	6	650	955	775	132	0.5520	0.8312	0.4480	0.4050	0.8545
N_aberrations	3	6	584	738	987	197	0.4278	0.7478	0.5722	0.4418	0.8336

N_aberrations	4	6	519	576	1128	259	0.3380	0.6671	0.6620	0.4740	0.8133
N_aberrations	5	6	451	445	1239	327	0.2643	0.5797	0.7357	0.5033	0.7912
N_aberrations	6	6	382	347	1314	394	0.2089	0.4923	0.7911	0.5240	0.7693
UCL	0.05	6	749	1331	394	32	0.7716	0.9590	0.2284	0.3601	0.9249
UCL	0.1	6	745	1312	413	36	0.7606	0.9539	0.2394	0.3622	0.9198
UCL	0.15	6	742	1241	484	39	0.7194	0.9501	0.2806	0.3742	0.9254
UCL	0.2	6	720	1160	565	61	0.6725	0.9219	0.3275	0.3830	0.9026
UCL	0.25	6	697	1054	671	84	0.6110	0.8924	0.3890	0.3981	0.8887
UCL	0.3	6	662	984	741	119	0.5704	0.8476	0.4296	0.4022	0.8616
UCL	0.35	6	633	924	801	148	0.5357	0.8105	0.4643	0.4066	0.8440
UCL	0.4	6	608	863	862	173	0.5003	0.7785	0.4997	0.4133	0.8329
UCL	0.45	6	595	800	925	186	0.4638	0.7618	0.5362	0.4265	0.8326
UCL	0.5	6	584	738	987	197	0.4278	0.7478	0.5722	0.4418	0.8336
UCL	0.55	6	541	684	1041	240	0.3965	0.6927	0.6035	0.4416	0.8126
UCL	0.6	6	520	629	1096	261	0.3646	0.6658	0.6354	0.4526	0.8077
UCL	0.65	6	493	554	1171	288	0.3212	0.6312	0.6788	0.4709	0.8026
UCL	0.7	6	455	498	1227	326	0.2887	0.5826	0.7113	0.4774	0.7901
UCL	0.75	6	437	426	1299	344	0.2470	0.5595	0.7530	0.5064	0.7906
UCL	0.8	6	404	373	1352	377	0.2162	0.5173	0.7838	0.5199	0.7820
UCL	0.85	6	388	324	1401	393	0.1878	0.4968	0.8122	0.5449	0.7809
UCL	0.9	6	358	284	1441	423	0.1646	0.4584	0.8354	0.5576	0.7731
UCL	0.95	6	344	260	1465	437	0.1507	0.4405	0.8493	0.5695	0.7702
