# Supplementary material for: Exploring Options for Syndromic Surveillance in Aquaculture: Outbreak Detection of Salmon Pancreas Disease Using Production Data from Norwegian Farms
Source: Transbound Emerg Dis. 2024 Apr 30;2024:9861677. doi: 10.1155/2024/9861677 (PMC12017065; doi:10.1155/2024/9861677)
Supplement: Supplementary 2 — Results of the cohorts' performance evaluation of the proposed syndromic surveillance system using various parameter values. TP = true positive, FP = false positive, TN = true negative, FN = false negative, Se = sensitivity, FAR = false alarm rate, PPV = positive predictive value, NPV = negative predictive value, time = timeliness, Q1 = 1st quantile, Q3 = 3rd quantile. [file 9861677.f2.pdf]

Parameter	Parameter setting	Production area	TP	FP	TN	FN	FAR	TPR(Se)	TNR(Sp)	PPV	NPV	Median time months	Q1 time months	Q3 time months
baseline.correction	FALSE	3	70	69	80	16	0.4631	0.8140	0.5369	0.5036	0.8333	1.00	-3.00	4.00
baseline.correction	TRUE	3	73	72	77	13	0.4832	0.8488	0.5168	0.5034	0.8556	1.00	-3.00	4.00
Model	1	3	73	81	68	13	0.5436	0.8488	0.4564	0.4740	0.8395	-1.00	-3.00	1.75
Model	2	3	79	85	64	7	0.5705	0.9186	0.4295	0.4817	0.9014	0.00	-3.00	2.00
Model	3	3	79	85	64	7	0.5705	0.9186	0.4295	0.4817	0.9014	1.00	-3.00	3.00
Model	4	3	79	86	63	7	0.5772	0.9186	0.4228	0.4788	0.9000	1.00	-3.00	3.00
Model	5	3	75	72	77	11	0.4832	0.8721	0.5168	0.5102	0.8750	1.00	-3.50	3.00
Model	6	3	73	72	77	13	0.4832	0.8488	0.5168	0.5034	0.8556	1.00	-3.00	4.00
Model	7	3	72	76	73	14	0.5101	0.8372	0.4899	0.4865	0.8391	1.00	-3.25	4.00
Model	8	3	72	74	75	14	0.4966	0.8372	0.5034	0.4932	0.8427	1.00	-3.25	4.00
Model	no_model	3	44	40	109	42	0.2685	0.5116	0.7315	0.5238	0.7219	4.50	0.75	7.00
MR_cutoff	0.003	3	86	130	19	0	0.8725	1.0000	0.1275	0.3981	1.0000	-3.00	-7.00	-1.00
MR_cutoff	0.004	3	86	123	26	0	0.8255	1.0000	0.1745	0.4115	1.0000	-3.00	-6.00	0.00
MR_cutoff	0.005	3	86	119	30	0	0.7987	1.0000	0.2013	0.4195	1.0000	-2.00	-6.00	0.00
MR_cutoff	0.006	3	85	114	35	1	0.7651	0.9884	0.2349	0.4271	0.9722	-2.00	-6.00	1.00
MR_cutoff	0.007	3	81	111	38	5	0.7450	0.9419	0.2550	0.4219	0.8837	-2.00	-5.00	1.00
MR_cutoff	0.008	3	81	105	44	5	0.7047	0.9419	0.2953	0.4355	0.8980	-2.00	-5.00	1.00
MR_cutoff	0.009	3	80	102	47	6	0.6846	0.9302	0.3154	0.4396	0.8868	-2.00	-4.00	1.25
MR_cutoff	0.01	3	79	97	52	7	0.6510	0.9186	0.3490	0.4489	0.8814	-1.00	-4.00	1.50
MR_cutoff	0.011	3	79	92	57	7	0.6174	0.9186	0.3826	0.4620	0.8906	-1.00	-4.00	1.50
MR_cutoff	0.012	3	79	86	63	7	0.5772	0.9186	0.4228	0.4788	0.9000	-1.00	-4.00	2.00
MR_cutoff	0.013	3	78	82	67	8	0.5503	0.9070	0.4497	0.4875	0.8933	0.00	-3.00	2.00
MR_cutoff	0.014	3	77	77	72	9	0.5168	0.8953	0.4832	0.5000	0.8889	1.00	-3.00	3.00
MR_cutoff	0.015	3	77	75	74	9	0.5034	0.8953	0.4966	0.5066	0.8916	1.00	-3.00	3.00
MR_cutoff	0.016	3	76	75	74	10	0.5034	0.8837	0.4966	0.5033	0.8810	1.00	-3.00	3.00
MR_cutoff	0.017	3	75	75	74	11	0.5034	0.8721	0.4966	0.5000	0.8706	1.00	-3.00	3.00
MR_cutoff	0.018	3	74	73	76	12	0.4899	0.8605	0.5101	0.5034	0.8636	1.00	-3.00	3.00
MR_cutoff	0.019	3	74	72	77	12	0.4832	0.8605	0.5168	0.5068	0.8652	1.00	-3.00	4.00
MR_cutoff	0.02	3	73	72	77	13	0.4832	0.8488	0.5168	0.5034	0.8556	1.00	-3.00	4.00
MR_cutoff	0.021	3	71	71	78	15	0.4765	0.8256	0.5235	0.5000	0.8387	1.00	-3.00	4.00
MR_cutoff	0.022	3	70	71	78	16	0.4765	0.8140	0.5235	0.4965	0.8298	1.00	-2.75	4.00

MR_cutoff	0.023	3	70	70	79	16	0.4698	0.8140	0.5302	0.5000	0.8316	1.00	-2.75	4.00
MR_cutoff	0.024	3	68	70	79	18	0.4698	0.7907	0.5302	0.4928	0.8144	1.00	-3.00	4.00
MR_cutoff	0.025	3	67	68	81	19	0.4564	0.7791	0.5436	0.4963	0.8100	1.00	-3.00	4.00
MR_cutoff	0.026	3	67	67	82	19	0.4497	0.7791	0.5503	0.5000	0.8119	1.00	-2.50	4.00
MR_cutoff	0.027	3	65	65	84	21	0.4362	0.7558	0.5638	0.5000	0.8000	1.00	-3.00	4.00
MR_cutoff	0.028	3	64	65	84	22	0.4362	0.7442	0.5638	0.4961	0.7925	1.50	-3.00	4.00
MR_cutoff	0.029	3	63	65	84	23	0.4362	0.7326	0.5638	0.4922	0.7850	2.00	-2.50	4.00
MR_cutoff	0.03	3	60	65	84	26	0.4362	0.6977	0.5638	0.4800	0.7636	1.50	-3.00	4.25
MR_cutoff	0.031	3	62	65	84	24	0.4362	0.7209	0.5638	0.4882	0.7778	2.00	-2.75	4.00
MR_cutoff	0.032	3	62	65	84	24	0.4362	0.7209	0.5638	0.4882	0.7778	2.00	-2.75	4.00
MR_cutoff	0.033	3	62	65	84	24	0.4362	0.7209	0.5638	0.4882	0.7778	2.00	-2.75	5.00
MR_cutoff	0.034	3	61	65	84	25	0.4362	0.7093	0.5638	0.4841	0.7706	2.00	-3.00	5.00
MR_cutoff	0.035	3	60	65	84	26	0.4362	0.6977	0.5638	0.4800	0.7636	2.00	-3.00	5.00
MR_cutoff	0.036	3	60	63	86	26	0.4228	0.6977	0.5772	0.4878	0.7679	2.00	-3.00	5.00
MR_cutoff	0.037	3	60	61	88	26	0.4094	0.6977	0.5906	0.4959	0.7719	2.00	-2.00	5.00
MR_cutoff	0.038	3	60	61	88	26	0.4094	0.6977	0.5906	0.4959	0.7719	2.00	-2.00	5.00
MR_cutoff	0.039	3	59	61	88	27	0.4094	0.6860	0.5906	0.4917	0.7652	2.00	-2.00	5.00
MR_cutoff	0.04	3	58	61	88	28	0.4094	0.6744	0.5906	0.4874	0.7586	2.00	-2.00	5.00
MR_cutoff	0.041	3	58	61	88	28	0.4094	0.6744	0.5906	0.4874	0.7586	2.00	-2.00	5.00
MR_cutoff	0.042	3	56	61	88	30	0.4094	0.6512	0.5906	0.4786	0.7458	2.00	-2.00	5.00
MR_cutoff	0.043	3	56	60	89	30	0.4027	0.6512	0.5973	0.4828	0.7479	2.00	-2.00	5.00
MR_cutoff	0.044	3	56	57	92	30	0.3826	0.6512	0.6174	0.4956	0.7541	2.00	-2.00	5.25
MR_cutoff	0.045	3	56	56	93	30	0.3758	0.6512	0.6242	0.5000	0.7561	2.00	-2.00	5.25
MR_cutoff	0.046	3	56	56	93	30	0.3758	0.6512	0.6242	0.5000	0.7561	2.00	-2.00	5.25
MR_cutoff	0.047	3	56	56	93	30	0.3758	0.6512	0.6242	0.5000	0.7561	2.00	-2.00	5.25
MR_cutoff	0.048	3	56	56	93	30	0.3758	0.6512	0.6242	0.5000	0.7561	2.00	-2.00	5.25
MR_cutoff	0.049	3	56	56	93	30	0.3758	0.6512	0.6242	0.5000	0.7561	2.00	-2.00	5.25
MR_cutoff	0.05	3	56	56	93	30	0.3758	0.6512	0.6242	0.5000	0.7561	2.00	-2.00	5.25
N_aberrations	1	3	86	146	23	0	0.8639	1.0000	0.1361	0.3707	1.0000	-4.00	-8.00	-2.00
N_aberrations	2	3	82	108	53	4	0.6708	0.9535	0.3292	0.4316	0.9298	-2.00	-5.00	1.00
N_aberrations	3	3	73	72	77	13	0.4832	0.8488	0.5168	0.5034	0.8556	1.00	-3.00	4.00
N_aberrations	4	3	59	56	81	26	0.4088	0.6941	0.5912	0.5130	0.7570	3.00	-0.50	6.00
N_aberrations	5	3	39	39	89	46	0.3047	0.4588	0.6953	0.5000	0.6593	3.00	0.00	6.00
N_aberrations	6	3	25	23	97	60	0.1917	0.2941	0.8083	0.5208	0.6178	5.00	1.00	7.00
UCL	0.05	3	86	148	1	0	0.9933	1.0000	0.0067	0.3675	1.0000	-6.00	-9.00	-2.00

UCL	0.1	3	86	147	2	0	0.9866	1.0000	0.0134	0.3691	1.0000	-6.00	-9.00	-2.00
UCL	0.15	3	86	147	2	0	0.9866	1.0000	0.0134	0.3691	1.0000	-6.00	-9.00	-2.00
UCL	0.2	3	86	142	7	0	0.9530	1.0000	0.0470	0.3772	1.0000	-5.00	-8.00	-1.00
UCL	0.25	3	86	135	14	0	0.9060	1.0000	0.0940	0.3891	1.0000	-4.00	-7.00	-1.00
UCL	0.3	3	86	127	22	0	0.8523	1.0000	0.1477	0.4038	1.0000	-3.00	-6.00	0.00
UCL	0.35	3	85	121	28	1	0.8121	0.9884	0.1879	0.4126	0.9655	-3.00	-6.00	0.00
UCL	0.4	3	84	115	34	2	0.7718	0.9767	0.2282	0.4221	0.9444	-2.00	-5.00	1.00
UCL	0.45	3	81	110	39	5	0.7383	0.9419	0.2617	0.4241	0.8864	-2.00	-5.00	1.75
UCL	0.5	3	79	98	51	7	0.6577	0.9186	0.3423	0.4463	0.8793	-2.00	-4.00	2.00
UCL	0.55	3	77	90	59	9	0.6040	0.8953	0.3960	0.4611	0.8676	-1.00	-4.00	2.00
UCL	0.6	3	75	75	74	11	0.5034	0.8721	0.4966	0.5000	0.8706	1.00	-3.00	3.00
UCL	0.65	3	73	72	77	13	0.4832	0.8488	0.5168	0.5034	0.8556	1.00	-3.00	4.00
UCL	0.7	3	69	69	80	17	0.4631	0.8023	0.5369	0.5000	0.8247	2.00	-3.00	4.00
UCL	0.75	3	61	65	84	25	0.4362	0.7093	0.5638	0.4841	0.7706	2.00	-3.00	5.00
UCL	0.8	3	54	59	90	32	0.3960	0.6279	0.6040	0.4779	0.7377	2.00	-2.75	5.00
UCL	0.85	3	51	51	98	35	0.3423	0.5930	0.6577	0.5000	0.7368	3.00	-2.00	5.50
UCL	0.9	3	48	49	100	38	0.3289	0.5581	0.6711	0.4948	0.7246	3.00	-0.25	6.00
UCL	0.95	3	47	46	103	39	0.3087	0.5465	0.6913	0.5054	0.7254	3.00	0.00	6.00
baseline.correction	FALSE	4	79	37	38	16	0.4933	0.8316	0.5067	0.6810	0.7037	1.00	-2.75	3.75
baseline.correction	TRUE	4	81	41	34	14	0.5467	0.8526	0.4533	0.6639	0.7083	1.00	-3.00	3.00
Model	1	4	79	37	38	16	0.4933	0.8316	0.5067	0.6810	0.7037	1.00	-4.00	3.00
Model	2	4	90	45	30	5	0.6000	0.9474	0.4000	0.6667	0.8571	1.00	-2.00	3.00
Model	3	4	90	45	30	5	0.6000	0.9474	0.4000	0.6667	0.8571	1.00	-2.00	4.00
Model	4	4	89	45	30	6	0.6000	0.9368	0.4000	0.6642	0.8333	1.00	-2.00	4.00
Model	5	4	83	43	32	12	0.5733	0.8737	0.4267	0.6587	0.7273	1.00	-3.00	3.00
Model	6	4	81	41	34	14	0.5467	0.8526	0.4533	0.6639	0.7083	1.00	-3.00	3.00
Model	7	4	81	39	36	14	0.5200	0.8526	0.4800	0.6750	0.7200	1.00	-2.00	4.00
Model	8	4	81	39	36	14	0.5200	0.8526	0.4800	0.6750	0.7200	1.00	-2.00	4.00
Model	no_model	4	52	13	62	43	0.1733	0.5474	0.8267	0.8000	0.5905	3.00	0.00	6.00
MR_cutoff	0.003	4	94	65	10	1	0.8667	0.9895	0.1333	0.5912	0.9091	-3.00	-6.00	0.00
MR_cutoff	0.004	4	93	63	12	2	0.8400	0.9789	0.1600	0.5962	0.8571	-2.00	-5.00	1.00
MR_cutoff	0.005	4	93	59	16	2	0.7867	0.9789	0.2133	0.6118	0.8889	-2.00	-5.00	1.00
MR_cutoff	0.006	4	92	58	17	3	0.7733	0.9684	0.2267	0.6133	0.8500	-1.50	-5.00	1.00
MR_cutoff	0.007	4	91	57	18	4	0.7600	0.9579	0.2400	0.6149	0.8182	-1.00	-5.00	2.00
MR_cutoff	0.008	4	91	55	20	4	0.7333	0.9579	0.2667	0.6233	0.8333	-1.00	-4.50	2.00

MR_cutoff	0.009	4	90	53	22	5	0.7067	0.9474	0.2933	0.6294	0.8148	0.00	-5.00	2.00
MR_cutoff	0.01	4	89	51	24	6	0.6800	0.9368	0.3200	0.6357	0.8000	0.00	-5.00	3.00
MR_cutoff	0.011	4	89	50	25	6	0.6667	0.9368	0.3333	0.6403	0.8065	0.00	-3.50	3.00
MR_cutoff	0.012	4	89	48	27	6	0.6400	0.9368	0.3600	0.6496	0.8182	1.00	-3.00	3.00
MR_cutoff	0.013	4	89	46	29	6	0.6133	0.9368	0.3867	0.6593	0.8286	1.00	-3.00	3.00
MR_cutoff	0.014	4	87	46	29	8	0.6133	0.9158	0.3867	0.6541	0.7838	1.00	-3.00	3.00
MR_cutoff	0.015	4	85	45	30	10	0.6000	0.8947	0.4000	0.6538	0.7500	1.00	-3.00	3.00
MR_cutoff	0.016	4	83	45	30	12	0.6000	0.8737	0.4000	0.6484	0.7143	1.00	-3.00	3.00
MR_cutoff	0.017	4	83	45	30	12	0.6000	0.8737	0.4000	0.6484	0.7143	1.00	-3.00	3.00
MR_cutoff	0.018	4	82	42	33	13	0.5600	0.8632	0.4400	0.6613	0.7174	1.00	-3.00	3.00
MR_cutoff	0.019	4	81	42	33	14	0.5600	0.8526	0.4400	0.6585	0.7021	1.00	-3.00	3.00
MR_cutoff	0.02	4	81	41	34	14	0.5467	0.8526	0.4533	0.6639	0.7083	1.00	-3.00	3.00
MR_cutoff	0.021	4	81	41	34	14	0.5467	0.8526	0.4533	0.6639	0.7083	1.00	-3.00	3.00
MR_cutoff	0.022	4	80	41	34	15	0.5467	0.8421	0.4533	0.6612	0.6939	1.00	-3.00	3.00
MR_cutoff	0.023	4	79	41	34	16	0.5467	0.8316	0.4533	0.6583	0.6800	1.00	-3.00	3.00
MR_cutoff	0.024	4	78	40	35	17	0.5333	0.8211	0.4667	0.6610	0.6731	1.00	-3.00	3.00
MR_cutoff	0.025	4	77	39	36	18	0.5200	0.8105	0.4800	0.6638	0.6667	1.00	-3.00	3.00
MR_cutoff	0.026	4	76	37	38	19	0.4933	0.8000	0.5067	0.6726	0.6667	1.00	-3.00	3.00
MR_cutoff	0.027	4	76	37	38	19	0.4933	0.8000	0.5067	0.6726	0.6667	1.00	-2.50	3.00
MR_cutoff	0.028	4	75	35	40	20	0.4667	0.7895	0.5333	0.6818	0.6667	1.00	-2.75	3.00
MR_cutoff	0.029	4	73	35	40	22	0.4667	0.7684	0.5333	0.6759	0.6452	1.00	-3.00	3.25
MR_cutoff	0.03	4	72	35	40	23	0.4667	0.7579	0.5333	0.6729	0.6349	1.00	-2.50	4.00
MR_cutoff	0.031	4	72	35	40	23	0.4667	0.7579	0.5333	0.6729	0.6349	1.00	-2.50	4.00
MR_cutoff	0.032	4	71	35	40	24	0.4667	0.7474	0.5333	0.6698	0.6250	1.00	-2.75	4.00
MR_cutoff	0.033	4	71	35	40	24	0.4667	0.7474	0.5333	0.6698	0.6250	1.00	-2.75	4.00
MR_cutoff	0.034	4	70	34	41	25	0.4533	0.7368	0.5467	0.6731	0.6212	1.00	-3.00	4.00
MR_cutoff	0.035	4	69	33	42	26	0.4400	0.7263	0.5600	0.6765	0.6176	1.00	-2.25	4.25
MR_cutoff	0.036	4	69	32	43	26	0.4267	0.7263	0.5733	0.6832	0.6232	1.00	-2.00	4.00
MR_cutoff	0.037	4	69	33	42	26	0.4400	0.7263	0.5600	0.6765	0.6176	1.00	-2.00	4.00
MR_cutoff	0.038	4	68	32	43	27	0.4267	0.7158	0.5733	0.6800	0.6143	1.00	-2.00	4.50
MR_cutoff	0.039	4	68	31	44	27	0.4133	0.7158	0.5867	0.6869	0.6197	1.00	-2.00	4.50
MR_cutoff	0.04	4	67	31	44	28	0.4133	0.7053	0.5867	0.6837	0.6111	1.00	-2.00	4.75
MR_cutoff	0.041	4	67	31	44	28	0.4133	0.7053	0.5867	0.6837	0.6111	1.00	-2.00	4.75
MR_cutoff	0.042	4	67	31	44	28	0.4133	0.7053	0.5867	0.6837	0.6111	1.00	-2.00	5.00
MR_cutoff	0.043	4	66	31	44	29	0.4133	0.6947	0.5867	0.6804	0.6027	1.00	-2.00	5.00

MR_cutoff	0.044	4	66	31	44	29	0.4133	0.6947	0.5867	0.6804	0.6027	1.00	-2.00	5.00
MR_cutoff	0.045	4	66	31	44	29	0.4133	0.6947	0.5867	0.6804	0.6027	1.00	-2.00	5.00
MR_cutoff	0.046	4	64	31	44	31	0.4133	0.6737	0.5867	0.6737	0.5867	1.00	-2.00	5.00
MR_cutoff	0.047	4	64	31	44	31	0.4133	0.6737	0.5867	0.6737	0.5867	1.00	-1.50	5.00
MR_cutoff	0.048	4	63	31	44	32	0.4133	0.6632	0.5867	0.6702	0.5789	1.50	-0.75	5.00
MR_cutoff	0.049	4	63	31	44	32	0.4133	0.6632	0.5867	0.6702	0.5789	1.50	-0.75	5.00
MR_cutoff	0.05	4	63	31	44	32	0.4133	0.6632	0.5867	0.6702	0.5789	1.50	-0.75	5.00
N_aberrations	1	4	96	77	15	0	0.8370	1.0000	0.1630	0.5549	1.0000	-4.00	-7.00	-1.00
N_aberrations	2	4	89	56	28	6	0.6667	0.9368	0.3333	0.6138	0.8235	-1.00	-4.50	2.00
N_aberrations	3	4	81	41	34	14	0.5467	0.8526	0.4533	0.6639	0.7083	1.00	-3.00	3.00
N_aberrations	4	4	67	33	38	27	0.4648	0.7128	0.5352	0.6700	0.5846	2.00	-1.00	5.00
N_aberrations	5	4	51	20	45	42	0.3077	0.5484	0.6923	0.7183	0.5172	2.00	-1.00	5.00
N_aberrations	6	4	44	14	47	49	0.2295	0.4731	0.7705	0.7586	0.4896	4.00	-1.00	6.00
UCL	0.05	4	95	75	0	0	1.0000	1.0000	0.0000	0.5588	NA	-5.00	-7.00	-1.00
UCL	0.1	4	95	75	0	0	1.0000	1.0000	0.0000	0.5588	NA	-5.00	-7.00	-1.00
UCL	0.15	4	95	74	1	0	0.9867	1.0000	0.0133	0.5621	1.0000	-4.00	-7.00	-1.00
UCL	0.2	4	95	73	2	0	0.9733	1.0000	0.0267	0.5655	1.0000	-4.00	-7.00	-1.00
UCL	0.25	4	95	71	4	0	0.9467	1.0000	0.0533	0.5723	1.0000	-4.00	-6.75	0.00
UCL	0.3	4	94	65	10	1	0.8667	0.9895	0.1333	0.5912	0.9091	-2.00	-5.50	1.00
UCL	0.35	4	92	60	15	3	0.8000	0.9684	0.2000	0.6053	0.8333	-2.00	-5.00	1.00
UCL	0.4	4	91	59	16	4	0.7867	0.9579	0.2133	0.6067	0.8000	-2.00	-5.00	1.00
UCL	0.45	4	91	54	21	4	0.7200	0.9579	0.2800	0.6276	0.8400	-1.00	-5.00	2.00
UCL	0.5	4	89	52	23	6	0.6933	0.9368	0.3067	0.6312	0.7931	-0.50	-4.25	3.00
UCL	0.55	4	89	48	27	6	0.6400	0.9368	0.3600	0.6496	0.8182	0.00	-3.00	3.00
UCL	0.6	4	84	45	30	11	0.6000	0.8842	0.4000	0.6512	0.7317	1.00	-3.00	3.00
UCL	0.65	4	81	41	34	14	0.5467	0.8526	0.4533	0.6639	0.7083	1.00	-3.00	3.00
UCL	0.7	4	78	36	39	17	0.4800	0.8211	0.5200	0.6842	0.6964	1.00	-2.00	4.00
UCL	0.75	4	74	32	43	21	0.4267	0.7789	0.5733	0.6981	0.6719	1.00	-2.00	4.00
UCL	0.8	4	69	30	45	26	0.4000	0.7263	0.6000	0.6970	0.6338	2.00	-1.00	5.00
UCL	0.85	4	61	26	49	34	0.3467	0.6421	0.6533	0.7011	0.5904	2.00	-1.00	6.00
UCL	0.9	4	56	22	53	39	0.2933	0.5895	0.7067	0.7179	0.5761	3.00	-0.75	6.00
UCL	0.95	4	54	18	57	41	0.2400	0.5684	0.7600	0.7500	0.5816	3.00	0.00	6.00
baseline.correction	FALSE	5	9	23	17	14	0.5750	0.3913	0.4250	0.2813	0.5484	1.00	0.00	5.00
baseline.correction	TRUE	5	13	28	12	10	0.7000	0.5652	0.3000	0.3171	0.5455	0.50	-1.75	3.50
Model	1	5	12	21	19	11	0.5250	0.5217	0.4750	0.3636	0.6333	-1.50	-3.25	4.25

Model	2	5	17	28	12	6	0.7000	0.7391	0.3000	0.3778	0.6667	-1.00	-3.00	2.00
Model	3	5	18	29	11	5	0.7250	0.7826	0.2750	0.3830	0.6875	-1.00	-2.50	3.50
Model	4	5	19	31	9	4	0.7750	0.8261	0.2250	0.3800	0.6923	0.50	-1.50	5.00
Model	5	5	12	27	13	11	0.6750	0.5217	0.3250	0.3077	0.5417	0.00	-3.00	1.00
Model	6	5	13	28	12	10	0.7000	0.5652	0.3000	0.3171	0.5455	0.50	-1.75	3.50
Model	7	5	15	27	13	8	0.6750	0.6522	0.3250	0.3571	0.6190	1.00	-0.50	4.50
Model	8	5	15	27	13	8	0.6750	0.6522	0.3250	0.3571	0.6190	1.00	-0.50	4.50
Model	no_model	5	2	4	36	21	0.1000	0.0870	0.9000	0.3333	0.6316	7.00	6.50	7.50
MR_cutoff	0.003	5	23	38	2	0	0.9500	1.0000	0.0500	0.3770	1.0000	-2.50	-5.50	0.25
MR_cutoff	0.004	5	23	37	3	0	0.9250	1.0000	0.0750	0.3833	1.0000	-2.50	-5.50	1.25
MR_cutoff	0.005	5	23	36	4	0	0.9000	1.0000	0.1000	0.3898	1.0000	-2.50	-5.00	1.25
MR_cutoff	0.006	5	22	36	4	1	0.9000	0.9565	0.1000	0.3793	0.8000	-1.00	-5.00	1.50
MR_cutoff	0.007	5	21	34	6	2	0.8500	0.9130	0.1500	0.3818	0.7500	-1.00	-4.00	2.75
MR_cutoff	0.008	5	19	33	7	4	0.8250	0.8261	0.1750	0.3654	0.6364	-1.00	-4.25	1.25
MR_cutoff	0.009	5	18	33	7	5	0.8250	0.7826	0.1750	0.3529	0.5833	-1.00	-4.50	1.00
MR_cutoff	0.01	5	18	33	7	5	0.8250	0.7826	0.1750	0.3529	0.5833	-1.00	-4.00	1.00
MR_cutoff	0.011	5	18	33	7	5	0.8250	0.7826	0.1750	0.3529	0.5833	-1.00	-3.50	1.00
MR_cutoff	0.012	5	18	33	7	5	0.8250	0.7826	0.1750	0.3529	0.5833	-1.00	-3.50	1.00
MR_cutoff	0.013	5	18	33	7	5	0.8250	0.7826	0.1750	0.3529	0.5833	-1.00	-3.50	1.00
MR_cutoff	0.014	5	18	33	7	5	0.8250	0.7826	0.1750	0.3529	0.5833	-1.00	-3.00	1.00
MR_cutoff	0.015	5	16	32	8	7	0.8000	0.6957	0.2000	0.3333	0.5333	-1.00	-3.00	1.00
MR_cutoff	0.016	5	16	32	8	7	0.8000	0.6957	0.2000	0.3333	0.5333	-1.00	-3.00	1.00
MR_cutoff	0.017	5	15	32	8	8	0.8000	0.6522	0.2000	0.3191	0.5000	-0.50	-3.25	1.00
MR_cutoff	0.018	5	15	32	8	8	0.8000	0.6522	0.2000	0.3191	0.5000	-0.50	-3.25	1.00
MR_cutoff	0.019	5	14	28	12	9	0.7000	0.6087	0.3000	0.3333	0.5714	0.00	-2.50	1.50
MR_cutoff	0.02	5	13	28	12	10	0.7000	0.5652	0.3000	0.3171	0.5455	0.50	-1.75	3.50
MR_cutoff	0.021	5	11	26	14	12	0.6500	0.4783	0.3500	0.2973	0.5385	1.00	-0.25	4.25
MR_cutoff	0.022	5	10	25	15	13	0.6250	0.4348	0.3750	0.2857	0.5357	1.00	-0.50	1.50
MR_cutoff	0.023	5	9	24	16	14	0.6000	0.3913	0.4000	0.2727	0.5333	0.50	-0.75	3.50
MR_cutoff	0.024	5	8	24	16	15	0.6000	0.3478	0.4000	0.2500	0.5161	1.00	0.00	5.00
MR_cutoff	0.025	5	8	24	16	15	0.6000	0.3478	0.4000	0.2500	0.5161	1.00	0.00	5.00
MR_cutoff	0.026	5	8	24	16	15	0.6000	0.3478	0.4000	0.2500	0.5161	1.00	0.00	5.00
MR_cutoff	0.027	5	8	24	16	15	0.6000	0.3478	0.4000	0.2500	0.5161	1.00	0.00	5.00
MR_cutoff	0.028	5	8	24	16	15	0.6000	0.3478	0.4000	0.2500	0.5161	1.00	0.00	5.00
MR_cutoff	0.029	5	8	22	18	15	0.5500	0.3478	0.4500	0.2667	0.5455	1.00	0.00	5.00

MR_cutoff	0.03	5	8	22	18	15	0.5500	0.3478	0.4500	0.2667	0.5455	1.00	0.00	5.00
MR_cutoff	0.031	5	8	22	18	15	0.5500	0.3478	0.4500	0.2667	0.5455	1.00	0.00	5.00
MR_cutoff	0.032	5	8	22	18	15	0.5500	0.3478	0.4500	0.2667	0.5455	1.00	0.00	5.00
MR_cutoff	0.033	5	8	21	19	15	0.5250	0.3478	0.4750	0.2759	0.5588	1.00	0.00	5.00
MR_cutoff	0.034	5	8	21	19	15	0.5250	0.3478	0.4750	0.2759	0.5588	1.00	0.00	5.00
MR_cutoff	0.035	5	8	21	19	15	0.5250	0.3478	0.4750	0.2759	0.5588	1.00	0.00	5.00
MR_cutoff	0.036	5	8	20	20	15	0.5000	0.3478	0.5000	0.2857	0.5714	2.50	0.00	5.50
MR_cutoff	0.037	5	8	20	20	15	0.5000	0.3478	0.5000	0.2857	0.5714	2.50	0.00	5.50
MR_cutoff	0.038	5	8	20	20	15	0.5000	0.3478	0.5000	0.2857	0.5714	2.50	0.00	5.50
MR_cutoff	0.039	5	8	20	20	15	0.5000	0.3478	0.5000	0.2857	0.5714	2.50	0.00	5.50
MR_cutoff	0.04	5	8	19	21	15	0.4750	0.3478	0.5250	0.2963	0.5833	2.50	0.00	5.50
MR_cutoff	0.041	5	8	19	21	15	0.4750	0.3478	0.5250	0.2963	0.5833	2.50	0.00	5.50
MR_cutoff	0.042	5	8	19	21	15	0.4750	0.3478	0.5250	0.2963	0.5833	2.50	0.00	5.50
MR_cutoff	0.043	5	8	18	22	15	0.4500	0.3478	0.5500	0.3077	0.5946	2.50	0.00	5.75
MR_cutoff	0.044	5	8	18	22	15	0.4500	0.3478	0.5500	0.3077	0.5946	2.50	0.00	5.75
MR_cutoff	0.045	5	8	19	21	15	0.4750	0.3478	0.5250	0.2963	0.5833	2.50	0.00	5.75
MR_cutoff	0.046	5	8	19	21	15	0.4750	0.3478	0.5250	0.2963	0.5833	2.50	0.00	5.75
MR_cutoff	0.047	5	8	19	21	15	0.4750	0.3478	0.5250	0.2963	0.5833	2.50	0.00	5.75
MR_cutoff	0.048	5	8	19	21	15	0.4750	0.3478	0.5250	0.2963	0.5833	2.50	0.00	5.75
MR_cutoff	0.049	5	8	19	21	15	0.4750	0.3478	0.5250	0.2963	0.5833	2.50	0.00	5.75
MR_cutoff	0.05	5	8	19	21	15	0.4750	0.3478	0.5250	0.2963	0.5833	2.50	0.00	5.75
N_aberrations	1	5	22	42	2	1	0.9545	0.9565	0.0455	0.3438	0.6667	-4.00	-7.00	-2.00
N_aberrations	2	5	20	37	5	3	0.8810	0.8696	0.1190	0.3509	0.6250	-1.00	-4.00	0.00
N_aberrations	3	5	13	28	12	10	0.7000	0.5652	0.3000	0.3171	0.5455	0.50	-1.75	3.50
N_aberrations	4	5	10	21	19	13	0.5250	0.4348	0.4750	0.3226	0.5938	2.00	1.00	6.50
N_aberrations	5	5	10	16	20	13	0.4444	0.4348	0.5556	0.3846	0.6061	3.00	2.00	7.50
N_aberrations	6	5	6	9	24	17	0.2727	0.2609	0.7273	0.4000	0.5854	6.50	3.25	9.75
UCL	0.05	5	23	40	0	0	1.0000	1.0000	0.0000	0.3651	NA	-4.00	-9.00	-0.75
UCL	0.1	5	23	40	0	0	1.0000	1.0000	0.0000	0.3651	NA	-4.00	-9.00	-0.75
UCL	0.15	5	23	40	0	0	1.0000	1.0000	0.0000	0.3651	NA	-4.00	-7.50	-0.75
UCL	0.2	5	23	40	0	0	1.0000	1.0000	0.0000	0.3651	NA	-4.00	-7.00	0.00
UCL	0.25	5	23	40	0	0	1.0000	1.0000	0.0000	0.3651	NA	-3.00	-7.00	0.00
UCL	0.3	5	23	39	1	0	0.9750	1.0000	0.0250	0.3710	1.0000	-2.50	-7.00	0.00
UCL	0.35	5	22	38	2	1	0.9500	0.9565	0.0500	0.3667	0.6667	-3.00	-5.00	-0.50
UCL	0.4	5	21	35	5	2	0.8750	0.9130	0.1250	0.3750	0.7143	-1.50	-5.00	0.75

UCL	0.45	5	19	34	6	4	0.8500	0.8261	0.1500	0.3585	0.6000	-1.00	-4.25	1.25
UCL	0.5	5	18	33	7	5	0.8250	0.7826	0.1750	0.3529	0.5833	-1.00	-4.50	1.00
UCL	0.55	5	17	33	7	6	0.8250	0.7391	0.1750	0.3400	0.5385	-0.50	-3.00	1.00
UCL	0.6	5	15	32	8	8	0.8000	0.6522	0.2000	0.3191	0.5000	-0.50	-3.25	1.00
UCL	0.65	5	13	28	12	10	0.7000	0.5652	0.3000	0.3171	0.5455	0.50	-1.75	3.50
UCL	0.7	5	8	23	17	15	0.5750	0.3478	0.4250	0.2581	0.5313	2.50	0.00	5.50
UCL	0.75	5	8	20	20	15	0.5000	0.3478	0.5000	0.2857	0.5714	2.50	0.00	5.50
UCL	0.8	5	6	16	24	17	0.4000	0.2609	0.6000	0.2727	0.5854	1.00	0.25	6.25
UCL	0.85	5	5	14	26	18	0.3500	0.2174	0.6500	0.2632	0.5909	2.00	1.00	8.00
UCL	0.9	5	4	11	29	19	0.2750	0.1739	0.7250	0.2667	0.6042	6.00	2.75	8.00
UCL	0.95	5	4	7	33	19	0.1750	0.1739	0.8250	0.3636	0.6346	6.00	2.75	8.00
baseline.correction	FALSE	6	73	26	71	35	0.2680	0.6759	0.7320	0.7374	0.6698	2.00	-0.50	6.00
baseline.correction	TRUE	6	77	36	61	31	0.3711	0.7130	0.6289	0.6814	0.6630	2.00	-1.00	5.00
Model	1	6	85	51	46	23	0.5258	0.7870	0.4742	0.6250	0.6667	0.50	-3.00	3.75
Model	2	6	92	55	42	16	0.5670	0.8519	0.4330	0.6259	0.7241	1.00	-2.00	5.00
Model	3	6	93	54	43	15	0.5567	0.8611	0.4433	0.6327	0.7414	1.00	-1.00	5.00
Model	4	6	91	49	48	17	0.5052	0.8426	0.4948	0.6500	0.7385	1.00	-1.00	5.00
Model	5	6	83	35	62	25	0.3608	0.7685	0.6392	0.7034	0.7126	1.00	-2.00	4.00
Model	6	6	77	36	61	31	0.3711	0.7130	0.6289	0.6814	0.6630	2.00	-1.00	5.00
Model	7	6	78	34	63	30	0.3505	0.7222	0.6495	0.6964	0.6774	2.00	-1.00	4.00
Model	8	6	78	32	65	30	0.3299	0.7222	0.6701	0.7091	0.6842	2.00	-1.00	4.00
Model	no_model	6	23	8	89	85	0.0825	0.2130	0.9175	0.7419	0.5115	5.00	1.00	10.00
MR_cutoff	0.003	6	104	86	11	4	0.8866	0.9630	0.1134	0.5474	0.7333	-2.00	-5.00	2.00
MR_cutoff	0.004	6	103	83	14	5	0.8557	0.9537	0.1443	0.5538	0.7368	-2.00	-5.00	2.00
MR_cutoff	0.005	6	100	72	25	8	0.7423	0.9259	0.2577	0.5814	0.7576	-1.00	-4.50	2.00
MR_cutoff	0.006	6	99	67	30	9	0.6907	0.9167	0.3093	0.5964	0.7692	-0.50	-4.00	2.75
MR_cutoff	0.007	6	96	64	33	12	0.6598	0.8889	0.3402	0.6000	0.7333	0.00	-4.00	3.00
MR_cutoff	0.008	6	95	59	38	13	0.6082	0.8796	0.3918	0.6169	0.7451	0.00	-3.00	3.25
MR_cutoff	0.009	6	93	55	42	15	0.5670	0.8611	0.4330	0.6284	0.7368	0.50	-2.75	3.00
MR_cutoff	0.01	6	91	53	44	17	0.5464	0.8426	0.4536	0.6319	0.7213	1.00	-3.00	3.00
MR_cutoff	0.011	6	91	49	48	17	0.5052	0.8426	0.4948	0.6500	0.7385	1.00	-2.25	4.00
MR_cutoff	0.012	6	91	48	49	17	0.4948	0.8426	0.5052	0.6547	0.7424	1.00	-2.00	4.00
MR_cutoff	0.013	6	89	46	51	19	0.4742	0.8241	0.5258	0.6593	0.7286	1.00	-2.00	4.00
MR_cutoff	0.014	6	87	43	54	21	0.4433	0.8056	0.5567	0.6692	0.7200	1.00	-2.00	4.00
MR_cutoff	0.015	6	86	41	56	22	0.4227	0.7963	0.5773	0.6772	0.7179	1.00	-2.00	4.00

MR_cutoff	0.016	6	83	38	59	25	0.3918	0.7685	0.6082	0.6860	0.7024	1.00	-2.00	4.00
MR_cutoff	0.017	6	82	38	59	26	0.3918	0.7593	0.6082	0.6833	0.6941	1.00	-2.00	4.00
MR_cutoff	0.018	6	79	37	60	29	0.3814	0.7315	0.6186	0.6810	0.6742	1.00	-2.00	5.00
MR_cutoff	0.019	6	79	36	61	29	0.3711	0.7315	0.6289	0.6870	0.6778	2.00	-1.75	5.00
MR_cutoff	0.02	6	77	36	61	31	0.3711	0.7130	0.6289	0.6814	0.6630	2.00	-1.00	5.00
MR_cutoff	0.021	6	77	35	62	31	0.3608	0.7130	0.6392	0.6875	0.6667	2.00	-1.00	5.00
MR_cutoff	0.022	6	77	34	63	31	0.3505	0.7130	0.6495	0.6937	0.6702	2.00	-1.00	5.00
MR_cutoff	0.023	6	76	33	64	32	0.3402	0.7037	0.6598	0.6972	0.6667	2.00	-1.00	5.00
MR_cutoff	0.024	6	76	31	66	32	0.3196	0.7037	0.6804	0.7103	0.6735	2.00	-1.00	5.00
MR_cutoff	0.025	6	75	28	69	33	0.2887	0.6944	0.7113	0.7282	0.6765	2.00	-1.00	5.00
MR_cutoff	0.026	6	73	28	69	35	0.2887	0.6759	0.7113	0.7228	0.6635	2.00	-1.00	5.00
MR_cutoff	0.027	6	73	28	69	35	0.2887	0.6759	0.7113	0.7228	0.6635	2.00	-1.00	5.00
MR_cutoff	0.028	6	72	27	70	36	0.2784	0.6667	0.7216	0.7273	0.6604	2.00	-1.00	5.00
MR_cutoff	0.029	6	71	26	71	37	0.2680	0.6574	0.7320	0.7320	0.6574	2.00	-1.00	6.00
MR_cutoff	0.03	6	70	25	72	38	0.2577	0.6481	0.7423	0.7368	0.6545	2.00	-1.00	6.00
MR_cutoff	0.031	6	67	25	72	41	0.2577	0.6204	0.7423	0.7283	0.6372	2.00	-1.00	5.00
MR_cutoff	0.032	6	67	23	74	41	0.2371	0.6204	0.7629	0.7444	0.6435	2.00	-1.00	5.00
MR_cutoff	0.033	6	65	24	73	43	0.2474	0.6019	0.7526	0.7303	0.6293	2.00	-1.00	5.00
MR_cutoff	0.034	6	63	19	78	45	0.1959	0.5833	0.8041	0.7683	0.6341	2.00	-1.00	5.00
MR_cutoff	0.035	6	63	20	77	45	0.2062	0.5833	0.7938	0.7590	0.6311	2.00	-1.00	5.00
MR_cutoff	0.036	6	63	19	78	45	0.1959	0.5833	0.8041	0.7683	0.6341	2.00	-1.00	5.00
MR_cutoff	0.037	6	63	19	78	45	0.1959	0.5833	0.8041	0.7683	0.6341	2.00	-1.00	5.00
MR_cutoff	0.038	6	62	18	79	46	0.1856	0.5741	0.8144	0.7750	0.6320	2.00	-1.00	5.00
MR_cutoff	0.039	6	62	18	79	46	0.1856	0.5741	0.8144	0.7750	0.6320	2.00	-1.00	5.00
MR_cutoff	0.04	6	63	19	78	45	0.1959	0.5833	0.8041	0.7683	0.6341	2.00	-1.00	5.00
MR_cutoff	0.041	6	62	18	79	46	0.1856	0.5741	0.8144	0.7750	0.6320	2.00	-1.00	5.00
MR_cutoff	0.042	6	61	19	78	47	0.1959	0.5648	0.8041	0.7625	0.6240	2.00	-1.00	5.00
MR_cutoff	0.043	6	61	18	79	47	0.1856	0.5648	0.8144	0.7722	0.6270	2.00	-1.00	5.00
MR_cutoff	0.044	6	61	18	79	47	0.1856	0.5648	0.8144	0.7722	0.6270	2.00	-1.00	5.00
MR_cutoff	0.045	6	61	16	81	47	0.1649	0.5648	0.8351	0.7922	0.6328	2.00	-1.00	5.00
MR_cutoff	0.046	6	59	16	81	49	0.1649	0.5463	0.8351	0.7867	0.6231	2.00	-1.00	5.00
MR_cutoff	0.047	6	58	16	81	50	0.1649	0.5370	0.8351	0.7838	0.6183	2.00	-1.00	5.00
MR_cutoff	0.048	6	57	16	81	51	0.1649	0.5278	0.8351	0.7808	0.6136	2.00	-1.00	5.00
MR_cutoff	0.049	6	56	16	81	52	0.1649	0.5185	0.8351	0.7778	0.6090	2.00	-1.75	5.00
MR_cutoff	0.05	6	55	16	81	53	0.1649	0.5093	0.8351	0.7746	0.6045	2.00	-2.00	5.00

N_aberrations	1	6	107	91	15	2	0.8585	0.9817	0.1415	0.5404	0.8824	-3.00	-6.00	0.00
N_aberrations	2	6	98	56	43	11	0.5657	0.8991	0.4343	0.6364	0.7963	-0.50	-5.00	3.00
N_aberrations	3	6	77	36	61	31	0.3711	0.7130	0.6289	0.6814	0.6630	2.00	-1.00	5.00
N_aberrations	4	6	58	22	68	49	0.2444	0.5421	0.7556	0.7250	0.5812	3.00	-0.50	6.00
N_aberrations	5	6	48	13	72	59	0.1529	0.4486	0.8471	0.7869	0.5496	4.00	1.00	7.00
N_aberrations	6	6	38	10	71	68	0.1235	0.3585	0.8765	0.7917	0.5108	5.00	1.50	11.00
UCL	0.05	6	107	97	0	1	1.0000	0.9907	0.0000	0.5245	0.0000	-5.00	-8.00	-1.00
UCL	0.1	6	107	97	0	1	1.0000	0.9907	0.0000	0.5245	0.0000	-5.00	-8.00	-1.00
UCL	0.15	6	107	97	0	1	1.0000	0.9907	0.0000	0.5245	0.0000	-5.00	-8.00	-1.00
UCL	0.2	6	107	96	1	1	0.9897	0.9907	0.0103	0.5271	0.5000	-4.00	-7.00	0.00
UCL	0.25	6	107	94	3	1	0.9691	0.9907	0.0309	0.5323	0.7500	-3.00	-6.00	0.00
UCL	0.3	6	104	89	8	4	0.9175	0.9630	0.0825	0.5389	0.6667	-2.00	-6.00	1.00
UCL	0.35	6	102	82	15	6	0.8454	0.9444	0.1546	0.5543	0.7143	-2.00	-5.00	1.75
UCL	0.4	6	99	76	21	9	0.7835	0.9167	0.2165	0.5657	0.7000	-1.50	-5.00	2.00
UCL	0.45	6	96	67	30	12	0.6907	0.8889	0.3093	0.5890	0.7143	-1.00	-4.00	2.00
UCL	0.5	6	95	55	42	13	0.5670	0.8796	0.4330	0.6333	0.7636	0.00	-3.00	3.00
UCL	0.55	6	91	44	53	17	0.4536	0.8426	0.5464	0.6741	0.7571	1.00	-2.00	4.00
UCL	0.6	6	83	40	57	25	0.4124	0.7685	0.5876	0.6748	0.6951	1.00	-2.00	4.00
UCL	0.65	6	77	36	61	31	0.3711	0.7130	0.6289	0.6814	0.6630	2.00	-1.00	5.00
UCL	0.7	6	73	28	69	35	0.2887	0.6759	0.7113	0.7228	0.6635	3.00	-0.50	6.00
UCL	0.75	6	64	20	77	44	0.2062	0.5926	0.7938	0.7619	0.6364	2.00	-1.00	5.00
UCL	0.8	6	53	17	80	55	0.1753	0.4907	0.8247	0.7571	0.5926	2.00	-1.00	5.50
UCL	0.85	6	44	13	84	64	0.1340	0.4074	0.8660	0.7719	0.5676	3.00	-1.00	8.00
UCL	0.9	6	33	12	85	75	0.1237	0.3056	0.8763	0.7333	0.5313	3.00	-0.75	9.50
UCL	0.95	6	27	11	86	81	0.1134	0.2500	0.8866	0.7105	0.5150	3.50	1.00	8.50
